# Mechanisms of Loss of Functions of Human Angiogenin Variants Implicated in Amyotrophic Lateral Sclerosis

**DOI:** 10.1371/journal.pone.0032479

**Published:** 2012-02-27

**Authors:** Aditya K. Padhi, Hirdesh Kumar, Suhas V. Vasaikar, Bhyravabhotla Jayaram, James Gomes

**Affiliations:** 1 School of Biological Sciences, Indian Institute of Technology Delhi, Hauz Khas, New Delhi, India; 2 Department of Chemistry, Indian Institute of Technology Delhi, Hauz Khas, New Delhi, India; 3 Supercomputing Facility for Bioinformatics and Computational Biology, Indian Institute of Technology Delhi, Hauz Khas, New Delhi, India; University of Regensburg, Germany

## Abstract

**Background:**

Mutations in the coding region of angiogenin (*ANG*) gene have been found in patients suffering from Amyotrophic Lateral Sclerosis (ALS). Neurodegeneration results from the loss of angiogenic ability of ANG (protein coded by *ANG*). In this work, we performed extensive molecular dynamics (MD) simulations of wild-type ANG and disease associated ANG variants to elucidate the mechanism behind the loss of ribonucleolytic activity and nuclear translocation activity, functions needed for angiogenesis.

**Methodology/Principal Findings:**

MD simulations were carried out to study the structural and dynamic differences in the catalytic site and nuclear localization signal residues between WT-ANG (Wild-type ANG) and six mutants. Variants K17I, S28N, P112L and V113I have confirmed association with ALS, while T195C and A238G single nucleotide polymorphisms (SNPs) encoding L35P and K60E mutants respectively, have not been associated with ALS. Our results show that loss of ribonucleolytic activity in K17I is caused by conformational switching of the catalytic residue His114 by 99°. The loss of nuclear translocation activity of S28N and P112L is caused by changes in the folding of the residues ^31^RRR^33^ that result in the reduction in solvent accessible surface area (SASA). Consequently, we predict that V113I will exhibit loss of angiogenic properties by loss of nuclear translocation activity and L35P by loss of both ribonucleolytic activity and nuclear translocation activity. No functional loss was inferred for K60E. The MD simulation results were supported by hydrogen bond interaction analyses and molecular docking studies.

**Conclusions/Significance:**

Conformational switching of catalytic residue His114 seems to be the mechanism causing loss of ribonucleolytic activity and reduction in SASA of nuclear localization signal residues ^31^RRR^33^ results in loss of nuclear translocation activity in ANG mutants. Therefore, we predict that L35P mutant, would exhibit loss of angiogenic functions, and hence would correlate with ALS while K60E would not show any loss.

## Introduction

Amyotrophic Lateral Sclerosis (ALS) is a fatal neurodegenerative disorder. It is caused by selective destruction of motor neurons [1]. A combination of several factors including protein aggregation, mitochondrial dysfunction, oxidative stress, defective axonal transport, excitotoxicity and dysfunctional growth factor signaling have been linked to the onset of ALS. In the later stages of the disease, paralysis sets in and death ensues due to respiratory failure. Approximately 10% of the cases are inherited in an autosomal dominant manner while 90% are sporadic. Among the catalogued ALS-like motor neuron diseases, mutations in *SOD1* (ALS1), *FUS/TLS* (ALS6), *VAPB* (ALS8), *ANG* (ALS9), *TARDBP* (ALS10), *FIG4* (ALS11) and a hexanucleotide-repeat expansion (GGGGCC) in the C9ORF72 cause adult onset neurodegenerative disorder [2]–[4]. Deletion of the hypoxia-response element in the vascular endothelial growth factor (*VEGF*) promoter also caused adult onset motor neuron degeneration similar to ALS in mice [5]. However, since mutations in *VEGF* have not been found in ALS patients, *VEGF* polymorphisms may be considered as a risk factor in some populations [6]. Among the ALS types that portray the characteristic adult onset neurodegenerative disorders, about one-fifth of the familial cases are attributed to missense mutations in the gene that encodes SOD1. Mutations in *SOD1* gene cause ALS through a toxic gain of function and not due to an impairment of its antioxidant function [7] and hence *SOD1* mimetics may not lead to an effective therapy. This and the fact that most reported cases of ALS are sporadic, underscores the importance of studying other gene mutations in detail.

Among other genes, heterozygous missense mutations in *ANG* have been associated with ALS [2]. ANG, a 123 amino acid single chain polypeptide (14.1 kDa), is strongly expressed in both endothelial cells and motor neurons in prenatal and adult spinal cord of humans. It influences the physiology and health of motor neurons by stimulating angiogenesis, neurite outgrowth and path-finding, and protects motor neurons under hypoxia [8], [9]. ANG maintains normal vasculature and thereby protects motor neurons from various stress conditions. Wu et al. [8] have shown using angiogenesis, ribonucleolysis, and nuclear translocation assays that ANG mutations identified in ALS patients are associated with functional loss of angiogenic activity. Baker et al. [10] and Cruts et al. [11] have also observed null mutations in another angiogenic protein, progranulin (PGRN), in frontotemporal dementia (FTD) patients. Mutations of *PGRN* gene were also reported in ALS patients [12]. Since compromised angiogenic activity appears to play a pivotal role in ALS progression, a study of the effect of selected mutations on the function of ANG may help in defining a better therapy.

**Figure 1:**
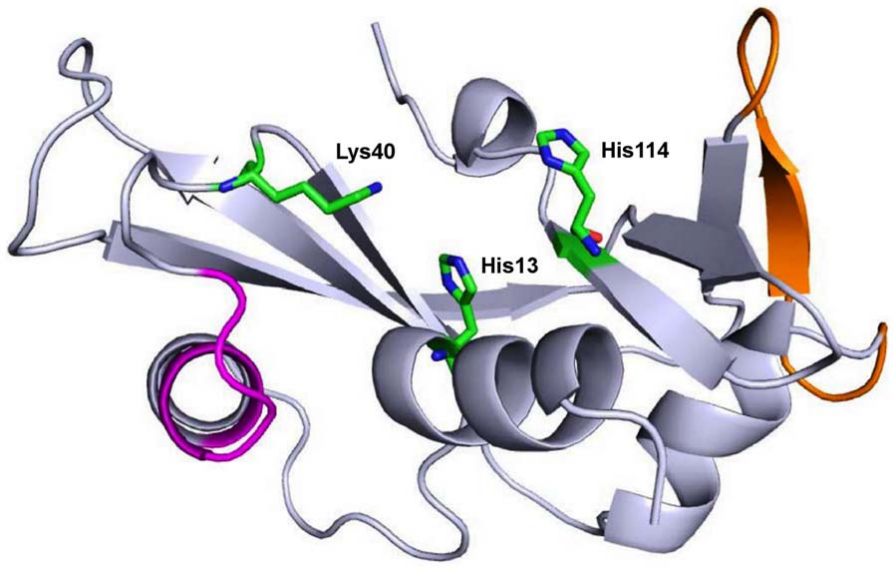
Cartoon representation of X-ray structure for Human Angiogenin (PDB code: 1B1I). Cartoon representation of the structure of Human Angiogenin (PDB entry 1B1I) showing its functional sites; catalytic triad residues are represented as stick models, nuclear localization signal is represented in magenta color and receptor binding site is represented in orange color. Figure produced using PyMOL [53].

ANG executes its essential functions via three functional sites (Figure 1). The first functional site, comprising the catalytic triad His13, Lys40 and His114, is responsible for ribonucleolytic activity. The second functional site consists of the nuclear localization signal ^29^IMRRRGL^35^, which resides on the surface of ANG and facilitates its translocation into nucleolus. In endothelial cells and motor neurons, ANG undergoes nuclear translocation, binds to the promoter region of ribosomal DNA and helps in ribosome biogenesis, protein translation and cell proliferation by stimulating rRNA transcription. The third functional site is the receptor-binding site ^60^NKNGNPHREN^68^, which is responsible for the binding of ANG to the endothelial cells, motor neurons and induces second messenger responses. Recent experimental studies have shown that mutations in ANG result in the loss of either ribonucleolytic activity, nuclear translocation activity or both and any single loss of either of these functions leads to the complete loss of angiogenic function which in turn causes ALS [8]. More than 15 *ANG* mutations have been associated with ALS of which 10 have been studied in detail [8], [9], [13], [14]–[20]. However, the molecular mechanism behind the functional loss of ANG due to these mutations is not completely understood.

**Figure 2:**
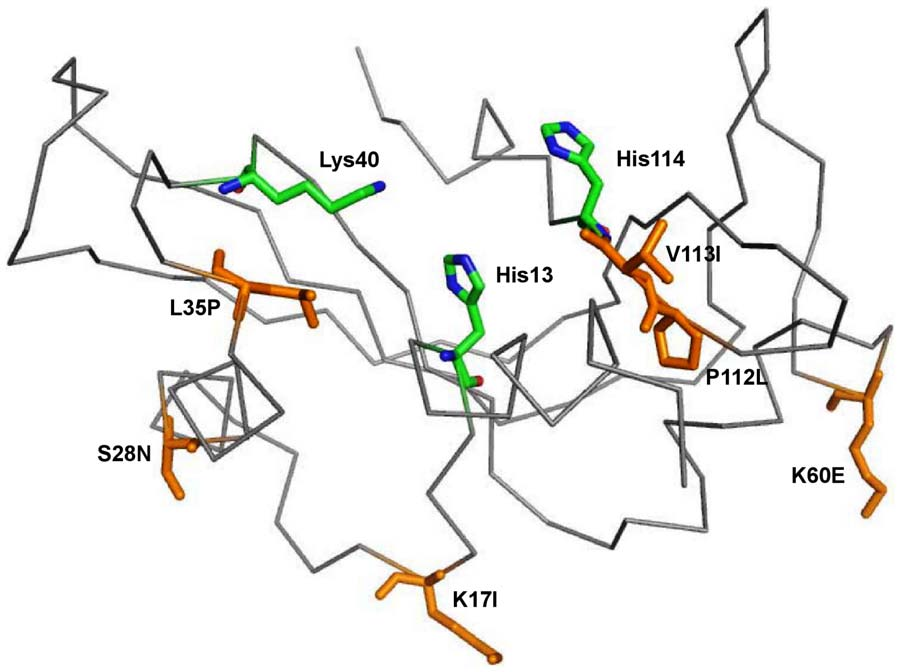
Ribbon representation of mutational sites in Human Angiogenin. Ribbon representation of structure of Human Angiogenin (PDB entry 1B1I) with mutational sites; mutations are labeled and represented as stick models in orange color. Figure produced using PyMOL [53].

In order to explain how these mutations resulted in the loss of either ribonucleolytic activity or nuclear translocation activity, or both, we have conducted a series of molecular dynamics (MD) simulations including all structurally different mutant forms that have complete ribonucleolytic and nuclear translocation activity information, except those near the catalytic site, so that our MD simulation results can be validated [8]: (i) K17I which results in the loss of ribonucleolytic activity, (ii) S28N which has only 9% of ribonucleolytic activity and no nuclear translocation activity, and (iii) P112L which results in partial ribonucleolytic activity and complete loss of nuclear translocation activity. We also studied the V113I variant which is prevalent among Italian patients [16]. In addition, our study included two missense SNPs, T195C and A238G at the gene level encoding L35P and K60E mutants respectively, not yet clinically correlated with ALS [21]. We performed 50 ns duration MD simulations of the WT-ANG, disease associated K17I, S28N, P112L and V113I variants and L35P and K60E mutants with AMBER 10 software suite. The sites of these mutations are shown in Figure 2.

This is the first study using MD simulations that presents an explanation for the loss of functions observed in ANG mutations. Our MD simulations demonstrate that a possible molecular mechanism may involve a change in conformation of the catalytic triad residue His114 resulting in the loss of ribonucleolytic activity. Simulation results confirm structural instability of ANG variants as reported in experimental studies. Further, we predict that L35P mutant may be responsible for causing ALS by loss of ribonucleolytic activity as well as nuclear translocation activity while K60E may be passive.

## Methods

### System setup

The crystal structure of human angiogenin (PDB: 1B1I) was used as the starting point [22]. The hetero atoms (crystallographic waters and cofactor, CIT) were removed from the structure before simulation. WT-ANG has three disulfide bonds Cys^26^–Cys^81^, Cys^39^–Cys^91^ and Cys^57^–Cys^107^ which were processed according to AMBER protocols. To model the starting structures of mutants K17I, S28N, P112L, L35P, K60E and V113I for which crystal structures do not exist, we mutated the corresponding residues *in silico* by replacing the target residues with the desired amino acids, keeping the secondary structures intact. All hydrogen atoms were added using the Xleap tool of AMBER 10 [23]. The system was solvated in an octahedral box of TIP3P water with ∼10 Å between the protein surface and the box boundary [24]. The free protein was neutralized by adding Cl^−^ counter ions for individual models. The SANDER module of AMBER 10 package was used for all the MD simulations.

### Molecular Dynamics Simulations

Simulations were initiated using the model of WT-ANG, K17I, S28N, P112L, L35P, K60E and V113I mutants of ANG and each simulation was performed for 50 ns in the isothermal isobaric ensemble (NPT). All simulations were performed according to the standard protocols, which consists of energy minimization, followed by gradual heating of the system. Each system was initially minimized employing 2500 steps of steepest descent followed by 1000 steps conjugate gradient minimization. Topology and parameter files for the protein were generated using “ff99SB” force field [25]. Each system was then gradually heated from 0 to 300 K in 200 ps followed by constant temperature equilibration at 300 K for 1000 ps. Following the equilibration phase, 50 ns MD simulations were carried out with periodic boundary conditions in the NPT ensemble, at a temperature of 300 K with Berendsen temperature coupling and a constant pressure of 1 atm with isotropic molecule-based scaling [26]. SHAKE algorithm was applied to fix all covalent bonds containing hydrogen atoms [27]. A 10 Å cutoff was applied to treat nonbonding interactions such as short-range electrostatics and van der Waals interactions, while the particle-mesh-Ewald (PME) method was used for long-range electrostatic interactions [28]. The coordinates of the trajectory was sampled every 1 ps for analysis of the energy stabilization and the root mean square deviations (RMSD) of the protein. The analyses of MD simulations were carried out by PTRAJ module of AMBER 10. MD simulations were performed on a 320 processor SUN Microsystems cluster at the Supercomputing Facility (http://www.scfbio-iitd.res.in) of Indian Institute of Technology Delhi.

### Molecular Docking

AutoDock 4.2 suite was used as molecular docking tool in order to carry out the docking simulations [29], [30]. Snapshots were generated from the MD trajectories for the native and altered His114 conformations and these structures were used for docking studies. The ligand molecule NCI-65828, which is an inhibitor of ribonucleolytic activity of ANG, was retrieved from NCBI-PubChem Compound database [PubChem: 5351857]. Docking logs were analyzed in the graphical user interface of ADT 1.5.4 [31]. Water molecules and Cl^−^ counter ions were cleaned off from the protein structure before docking. H-atoms were added to the target protein for correct ionization and tautomeric states of amino acids and the non-polar hydrogens were then merged. Kollman united atom charges and solvation parameters were assigned to the protein. Gasteiger charges were assigned to the ligand. The structures so obtained were converted to PDBQT format in ADT. The Lamarckian Genetic Algorithm was used with a population size of 150 dockings and 2.5 million energy evaluations were used in the docking experiments. All other parameters, such as crossover rate and mutation rate, were run with default settings. The grid size for specifying the search space was set at 80×80×80 centered on the macromolecule with a default grid point spacing of 0.375 Å. Pre-calculated grid maps, which store grids of interaction energy based on the interaction of the ligand atom probes with receptor target, were obtained using AutoGrid. The results were clustered into bins of similar conformations based on root mean square deviation (RMSD) and orientation.

The binding energies were also calculated using ParDOCK which is an all atom energy based Monte Carlo docking protocol [32]–[34]. Docking using ParDOCK requires a reference complex (target protein bound to a reference ligand) and a candidate molecule along with specific mention of the centre of mass of the cavity on which the ligand is to be docked. The active sites were predicted using Active Site Prediction server (http://www.scfbio-iitd.res.in/dock/ActiveSite.jsp), which computes cavities in the protein. The cavity in close proximity to His114 was used for docking the compound NCI-65828.

### Solvent accessible surface area

Solvent-accessible surface area (SASA) of nuclear localization signal residues ^31^RRR^33^ was calculated for the entire simulation period using SurfVol, a Plug-in for Visual Molecular Dynamics (VMD) software (version 1.8.7) [35] for measuring the surface area of proteins (http://www.compbiochem.org/Software/SurfVol/Home.html). The box used for the surface area calculation was centered on residues of the nuclear localization signal. For the seven cases studied in this work, SASA was calculated using a probe radius of 1.4 Å.

## Results

The objective of our simulation study was to determine the underlying cause for the loss of ribonucleolytic activity and nuclear translocation activity of ANG reported in ALS patients, and predict the role of certain SNPs reported in *ANG* but not yet clinically correlated in ALS patients. We observed the following steps to achieve this goal. The WT-ANG was simulated first and the results were compared with the crystal structure. This was followed by simulations of known disease associated variants K17I, S28N and P112L. Finally, simulations were performed for L35P, K60E and V113I mutants. The results of MD simulations have been supported with hydrogen-bond interaction analyses and docking studies of an inhibitor to the active site. Solvent accessible surface areas (SASA) were determined over the MD trajectories to understand changes in nuclear translocation activity.

### MD Simulations

The reliability of the system setup for performing the MD simulation was first evaluated using WT-ANG crystal structure as the basis because the crystal structures of the mutants were not available. The simulated structure of WT-ANG, obtained by averaging of all the frames in the 50 ns period, was superposed on the crystal structure and the root mean square deviation (RMSD) was found to be 0.30 Å. The root mean square fluctuation (RMSF) values calculated from the B-factor for the crystal structure and that obtained from the MD simulation were compared and found to be in good agreement (see Figure S1). We also validated the 50 ns period by performing a simulation of WT-ANG in which His114 was rotated *a priori* by 99^o^. We observed that within 1 ns, His114 of WT-ANG acquires its native conformation (see Figure S2) and consequently 50 ns was used in all MD simulations. In addition, we captured the structures of K17I, S28N and L35P mutants when His114 changed its conformation by 99°. We then computationally mutated each site of mutation back to the WT-ANG sequence and carried out a fresh set of MD simulations with these altered structures. It was observed that these structures returned to their WT-ANG conformation within 1 ns and remained stable thereafter. These results validated the MD model, which was then used for subsequent simulation experiments and analyses.

**Figure 3:**
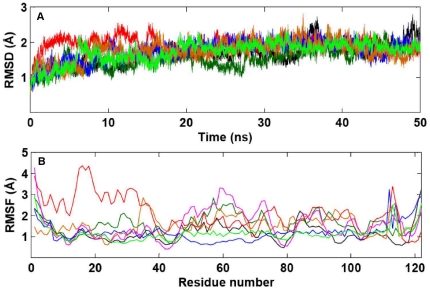
RMSD and RMSF profile for WT-ANG and mutants. (A) Control plots representing the stability of the models during the molecular dynamics run. The root mean square deviation (RMSD) of the backbone atoms from the equilibrated conformation (0 ns) is presented as a function of time. The RMSD time profiles for WT-ANG, K17I, S28N, P112L, L35P, K60E and V113I are shown in black, red, dark green, blue, orange, pink, and light green, respectively. (B) Root mean square fluctuation (RMSF) values of atomic positions computed for the backbone atoms are shown as a function of residue number. The RMSF values for WT-ANG, K17I, S28N, P112L, L35P, K60E and V113I are shown in black, red, dark green, blue, orange, pink, and light green, respectively.

MD simulations of all the mutants were performed for 50 ns to determine if there was any structural difference with WT-ANG that deserved attention. No major difference in the side chain orientations was observed. The RMSD and RMSF values obtained from the MD simulations of WT-ANG compared to K17I, S28N, P112L and V113I variants, and L35P and K60E mutants have been presented (Figure 3). It was observed that the RMSD of K17I was initially higher compared to the others. However, after 20 ns, the RMSDs in all cases are nearly similar (Figure 3A). The RMSF profiles were determined for the mutants and it was observed that in each case the highest fluctuation occurs at the point of mutation (Figure 3B). It was also observed that K17I mutation affects neighboring residues resulting in a higher RMSF up to residue 40. Further, the RMSF values for P112L and V113I were lower between residues 40 and 80 indicating a loss of flexibility caused by these mutations.

### Loss of Functions of ANG Mutants

#### Loss of Ribonucleolytic Activity

**Figure 4:**
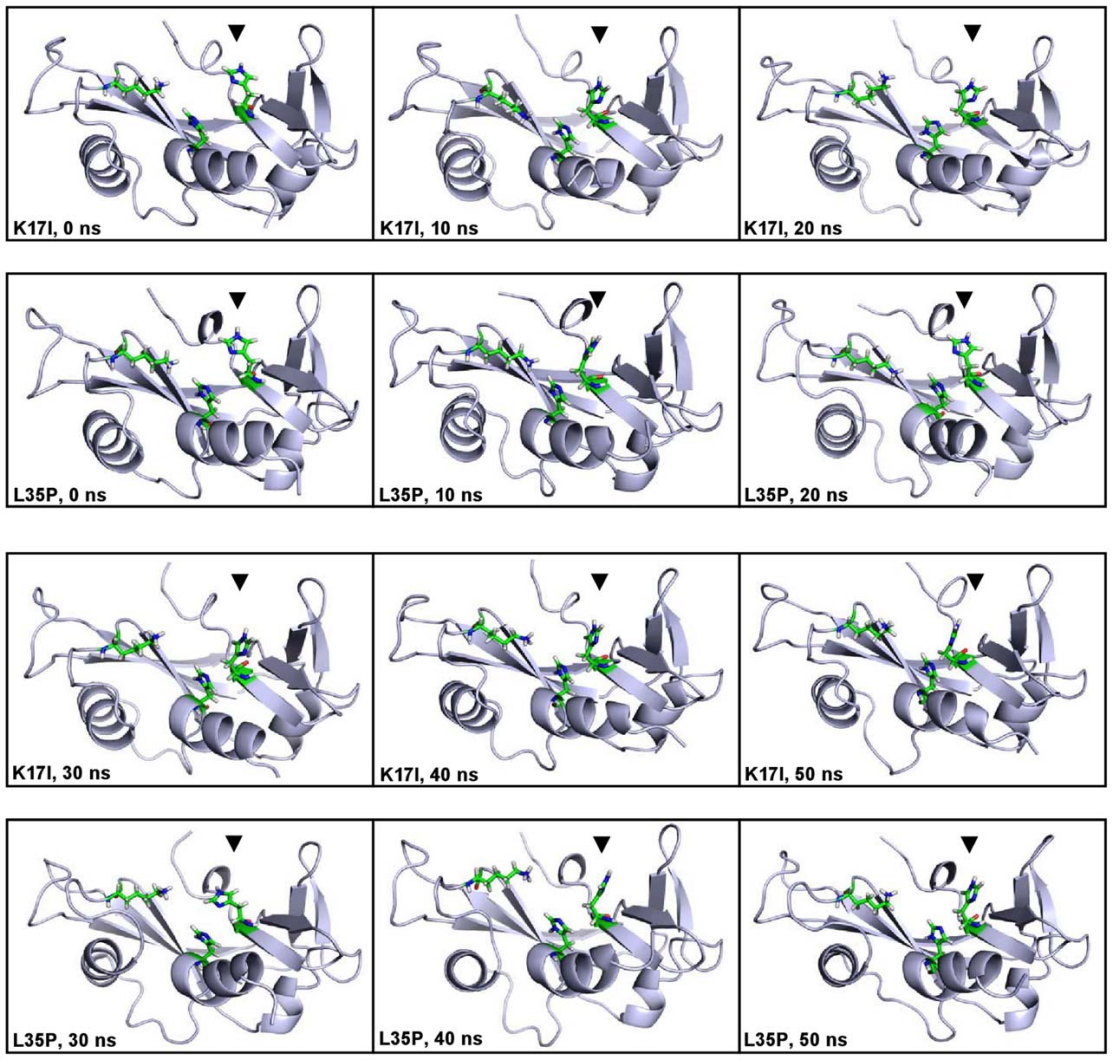
Conformational switching of catalytic residue His114 in K17I and L35P mutants. Orientation of the catalytic triad residue His114 at a regular interval of 10 ns during the MD simulations of K17I and L35P mutants. In these figures, T = 0 ns is the start of production phase post-equilibration. Figure produced using PyMOL [53].

**Figure 5:**
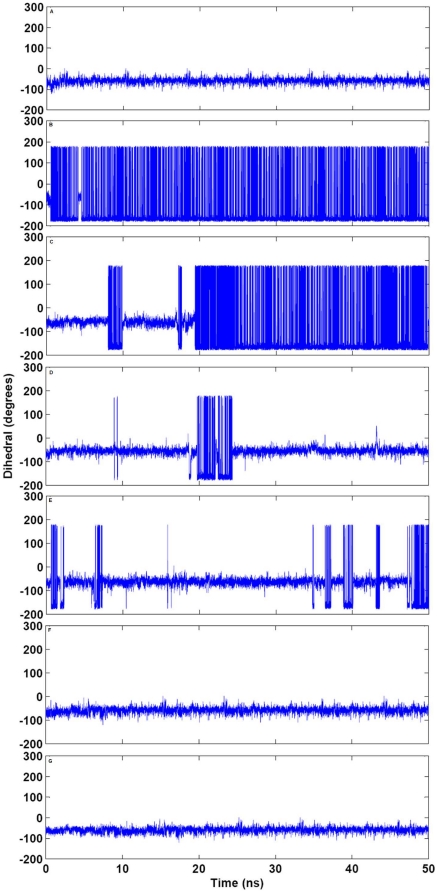
Computed change in HA-CA-CB-CG dihedral angle of His114 for WT-ANG and mutants. The HA-CA-CB-CG dihedral angle change of catalytic residue His114 computed as a function of time (A) WT-ANG (B) K17I (C) S28N (D) P112L (E) L35P (F) K60E and (G) V113I.

The catalytic triad of ANG consisting of Lys40, His13 and His114, confers ribonucleolytic activity on the protein. The catalytic triad was visualized using VMD [35] and it was observed that the orientation of His114 in the K17I variant changed significantly compared to WT-ANG. Snapshots of the catalytic triad at 10 ns intervals for K17I and L35P are given in Figure 4. For other mutants, the snapshots of catalytic triad at 10 ns intervals are given in Figures S3 and S4. The HA-CA-CB-CG dihedral angle of His114 was measured throughout the course of the simulation. It was observed that the dihedral angle changed from the mean −80° position of the WT-ANG and assumed a new dihedral orientation of −179° for K17I during most of the simulation period (Figures 5A and 5B). The L35P mutant also showed a similar change in conformation to −179° for the dihedral angle but for shorter durations (Figure 5E). The variants S28N and P112L showed changes in the dihedral angle after 20 ns (Figures 5C and 5D). The longer duration of dihedral change of His114 for S28N correlates with the reported observation that it retains 9% of ribonucleolytic activity and the shorter duration for P112L correlates with partial loss of activity [8]. The remaining mutants, K60E and V113I, did not exhibit this shift in dihedral and had profiles similar to WT-ANG (Figures 5F and 5G).

#### Hydrogen Bonding Interactions

The observation that only the His114 of the catalytic triad exhibited a significant change in the dihedral angle was further examined. Hydrogen-bond interactions from the site of mutation to His114 were analyzed to understand how a single mutation could cause the observed conformational switch.

**Figure 6:**
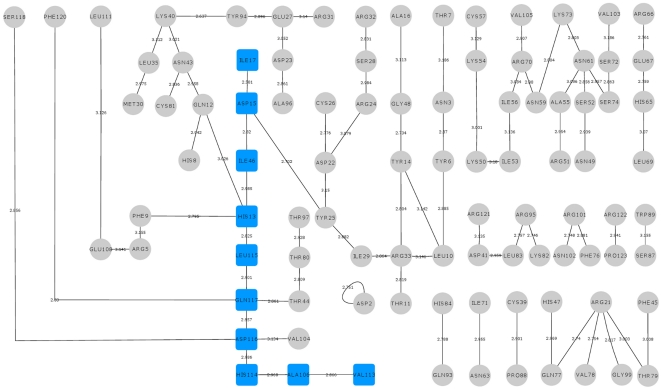
Hydrogen bond interaction network for K17I variant. The hydrogen bond interactions between contiguous amino acid residues based on a 3.2 Å cut-off has been presented here. The path leading from the site of mutation Ile17 to catalytic residue His114 has been shown in blue square boxes. Other hydrogen bond interactions are shown in grey circles. The bond length is given on the edge between the nodes of amino acid residues. The path is mediating through Leu115 which plays a role in His114 conformational switching.

**Figure 7:**
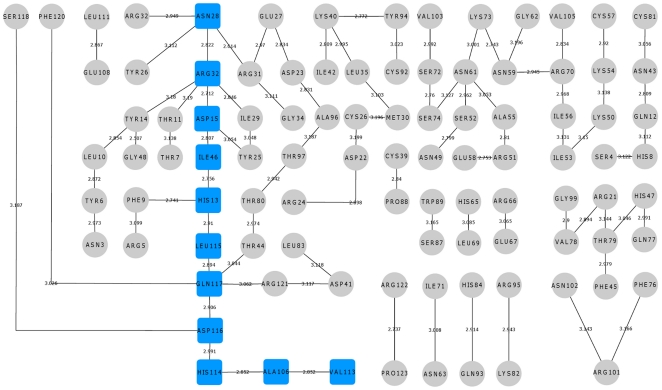
Hydrogen bond interaction network for S28N variant. The hydrogen bond interactions between contiguous amino acid residues based on a 3.2 Å cut-off has been presented here. The path leading from the site of mutation Asn28 to catalytic residue His114 has been shown in blue square boxes. Other hydrogen bond interactions are shown in grey circles. The bond length is given on the edge between the nodes of amino acid residues. The path is mediating through Leu115 which plays a role in His114 conformational switching.

**Figure 8:**
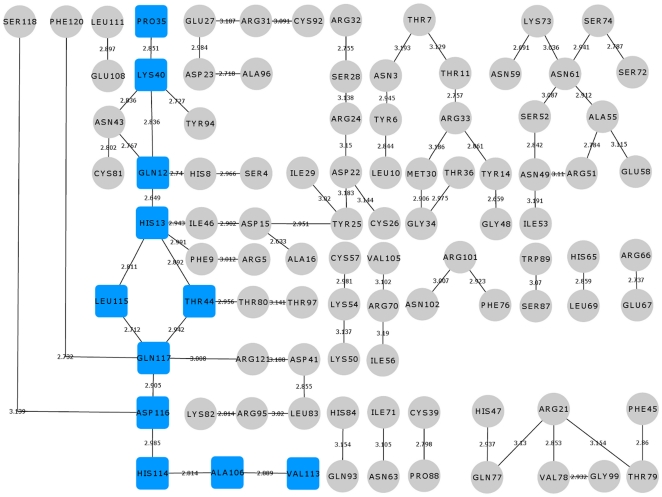
Hydrogen bond interaction network for L35P mutant. The hydrogen bond interactions between contiguous amino acid residues based on a 3.2 Å cut-off has been presented here. The paths leading from the site of mutation Pro35 to catalytic residue His114 have been shown in blue square boxes. Other hydrogen bond interactions are shown in grey circles. The bond length is given on the edge between the nodes of amino acid residues. One of the path is mediating through Leu115 which plays a role in His114 conformational switching and the other path is mediating through Thr44 similar to that of WT-ANG.

**Figure 9:**
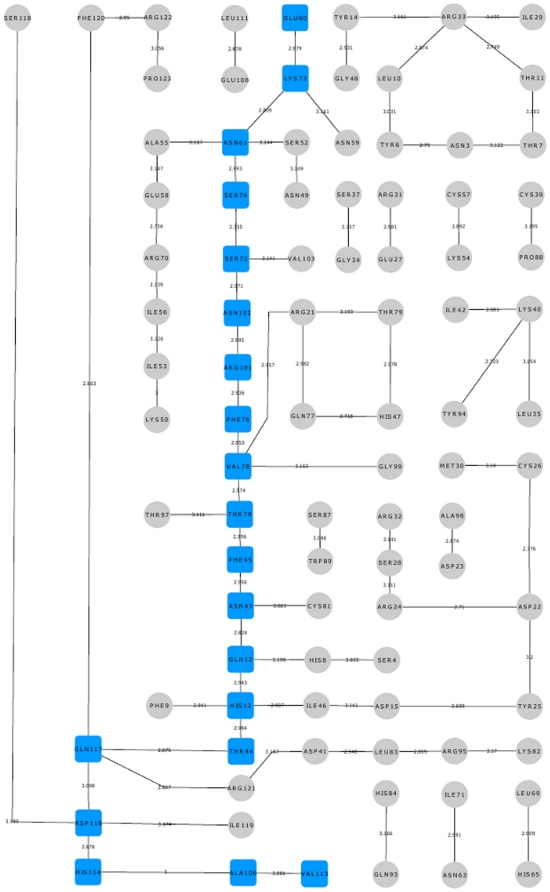
Hydrogen bond interaction network for K60E mutant. The hydrogen bond interactions between contiguous amino acid residues based on a 3.2 Å cut-off has been presented here. The path leading from the site of mutation Glu60 to catalytic residue His114 has been shown in blue square boxes. Other hydrogen bond interactions are shown in grey circles. The bond length is given on the edge between the nodes of amino acid residues. The path is mediating through Thr44 similar to that of WT-ANG and there is no conformational switching of His114.

**Figure 10:**
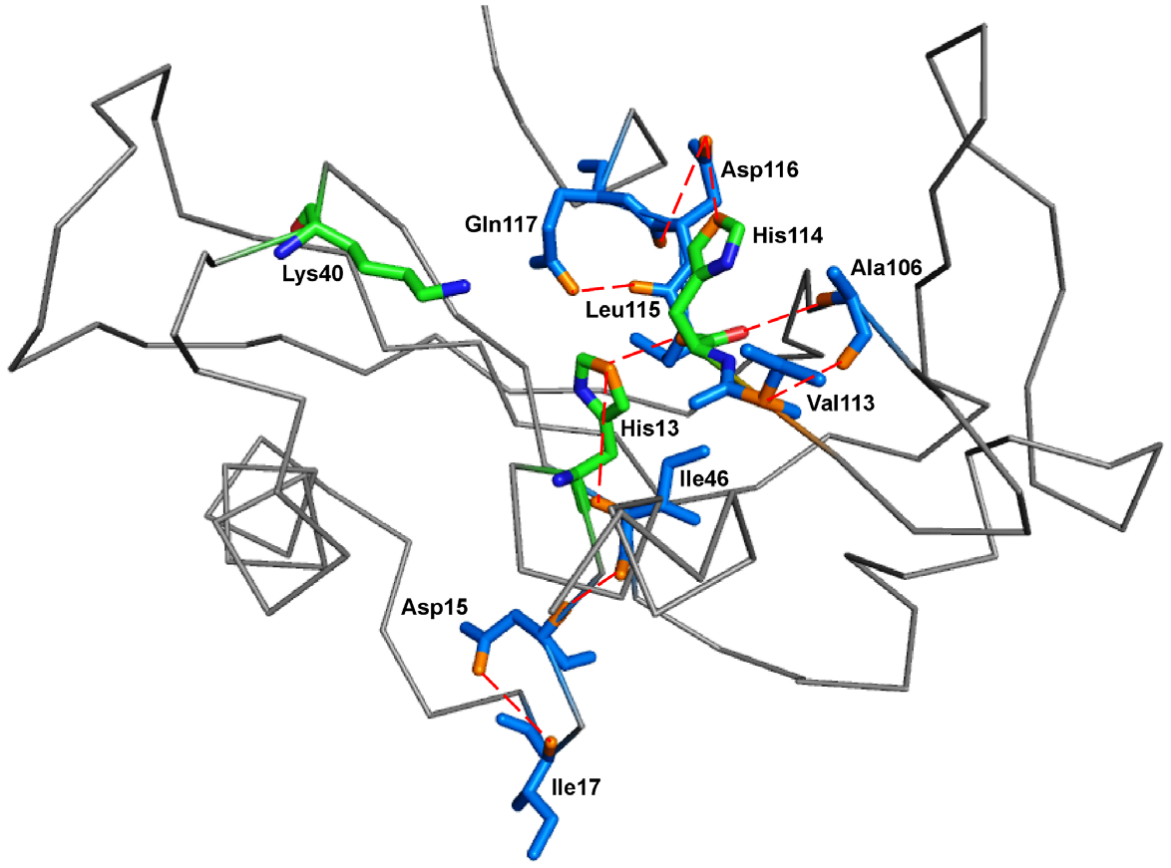
Residues interacting through hydrogen bonds from the site of mutation to His114 in K17I variant. Amino acid residues of K17I variant through which hydrogen bonds exert influence from the site of mutation on the catalytic site His114. The ribbon representation shows the shortest path traced by the contiguous amino acid sequence Ile17-Asp15-Ile46-His13-Leu115-Gln117-Asp116-His114-Ala106-Val113 and the hydrogen bond interactions. Residues have been shown in stick model in marine blue color. Catalytic triad residues have been shown as stick model and represented in green color. Hydrogen bonds between residues have been shown in red dashed-lines. Figure produced using PyMOL [53].

**Figure 11:**
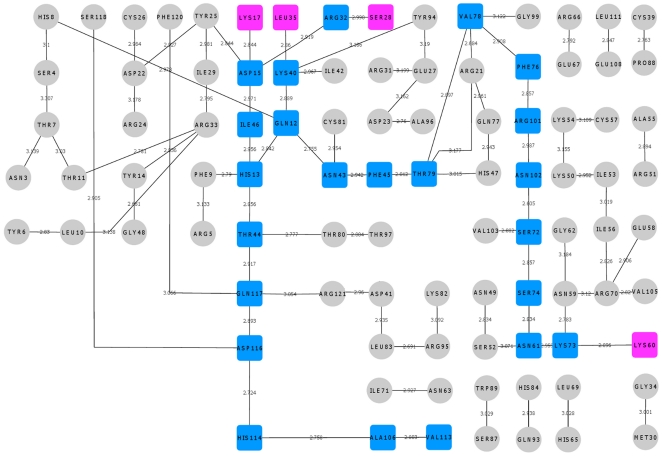
Hydrogen bond interaction network for WT-ANG. The hydrogen bond interactions between contiguous amino acid residues based on a 3.2 Å cut-off has been presented here. All hydrogen bond interaction paths up to catalytic residue His114 have been compared with the hydrogen bond interaction paths of other mutants. These hydrogen bond interactions are shown in blue square boxes. Other hydrogen bond interactions are shown in grey circles. The starting residues through which these paths led to His114 are shown in pink square boxes. The bond length is given on the edge between the nodes of amino acid residues. All the hydrogen bond interaction paths are mediating through Thr44 and there is no conformational switching of His114.

We observed that amino acids linked by hydrogen bonds were different for the mutants examined and hence used UCSF CHIMERA package [36] to identify the amino acid residues linked by hydrogen bonds (cut-off ≤3.2 Å). The hydrogen bond interaction network was visualized using Cytoscape [37] and all paths obtained were confirmed by adjacency matrix analysis using a MATLAB program. The paths starting from the site of mutation to His114 were identified for K17I, S28N, L35P and K60E mutants and presented (Figures 6, 7, 8, 9) but not for V113I and P112L variants because of their proximity to His114. In each case, we determined the shortest path leading from the site of mutation to His114. For example, the path for K17I was Ile17-Asp15-Ile46-His13-Leu115-Gln117-Asp116-His114-Ala106-Val113 as represented (Figures 6, 10) and the corresponding equivalent path in WT-ANG was Lys17-Asp15-Ile46-His13-Thr44-Gln117-Asp116-His114-Ala106-Val113 (Figure 11). Two amino acids following His114 have also been considered because these participate in the conformational switching of His114. We also captured snapshots at the point of conformational switching of His114 for various time instances in the case of mutants exhibiting loss of ribonucleolytic activity and found that the shortest path is always conserved.

**Figure 12:**
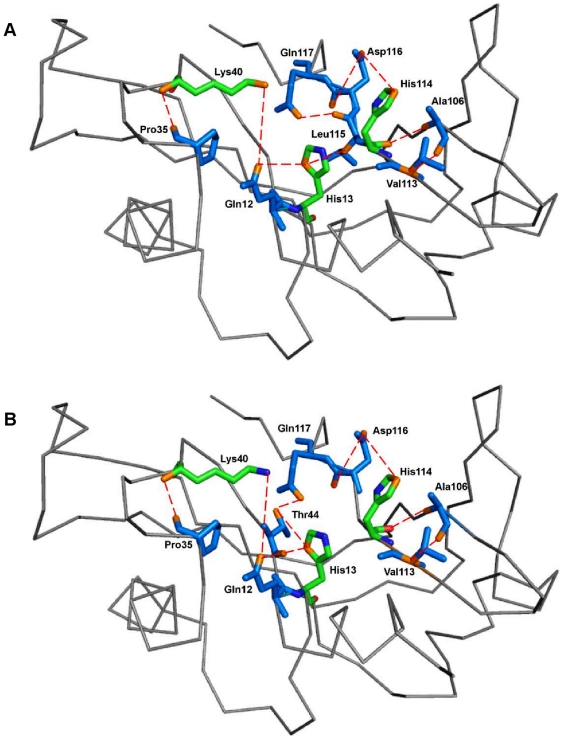
Residues interacting through hydrogen bonds from the site of mutation to His114 in L35P mutant. Amino acid residues of L35P mutant through which hydrogen bonds exert influence from the site of mutation on the catalytic site His114. There were two shortest paths identified. The first mediated through Leu115 and the second mediated through Thr44. Residues have been shown as stick models in marine blue color. Catalytic triad residues have been shown as stick model and represented in green color. Hydrogen bonds between residues have been shown in red dashed-lines. (A) The ribbon representation shows the path traced by the contiguous amino acid sequence Pro35-Lys40-Gln12-His13-Leu115-Gln117-Asp116-His114-Ala106-Val113 (B) The ribbon representation shows the path traced by the contiguous amino acid sequence Pro35-Lys40-Gln12-His13-Thr44-Gln117-Asp116-His114-Ala106-Val113. Figure produced using PyMOL [53].

Next, the hydrogen-bond occupancy for each adjacent pair of amino acids appearing in the path was determined for 50 ns (Table 1). We observed that hydrogen bonding between His114-Ala106 and Ala106-Val113 increases significantly in K17I mutant. Hydrogen bond occupancy for His114-Ala106 and Ala106-Val113 was 50.88% and 38.48% higher, compared with WT-ANG. In L35P, two possible paths were identified, the first mediated through Leu115 and the second through Thr44 (Figures 8, 12). A comparison of the Thr44 mediated path between L35P and WT-ANG showed 38.48% and 20.05% higher occupancies for His114-Ala106 and Ala106-Val113, which correlated with the shorter duration of dihedral angle shift.

**Table 1:** Percentage hydrogen bonding occupancy of adjacent pairs of amino acids for K17I, S28N, L35P and K60E in increasing order of ribonucleolytic activity.

H-bond Path	WT-ANG	K17I	H-bond Path	WT-ANG	L35P	
Donor-acceptor	%	%	Donor-acceptor	%	%	%
Lys17 (*Ile17*)-Asp15	10.22	0.55	Leu35 (*Pro35*)-Lys40	8.25	10.12	10.12
Asp15-Ile46	4.47	4.80	Lys40-Gln12	7.37	13.50	13.50
Ile46-His13	37.53	38.41	Gln12-His13	9.51	6.42	6.42
His13-Thr44 (Leu115)	21.74	18.61	His13-Thr44 (Leu115, Thr44)	21.74	17.28	16.59
Thr44 (Leu115)-Gln117	25.31	30.83	Thr44 (Leu115, Thr44)-Gln117	25.31	19.84	38.31
Gln117-Asp116	15.57	23.88	Gln117-Asp116	15.57	9.73	9.73
Asp116-His114	2.72	3.52	Asp116-His114	2.72	8.94	8.94
His114-Ala106	27.33	78.21	His114-Ala106	27.33	65.81	65.81
Ala106-Val113	43.10	81.58	Ala106-Val113	43.10	63.15	63.15

H-bond Path	WT-ANG	S28N	H-bond Path	WT-ANG	K60E		WT-ANG	K60E
Donor-acceptor	%	%	Donor-acceptor	%	%		%	%
Ser28 (*Asn28*)-Arg32	17.61	15.56	Lys60 (*Glu60*)-Lys73	3.55	1.38	*Phe45-Asn43	0.57	2.15
Arg32-Asp15	3.87	6.47	Lys73-Asn61	39.17	42.77	Asn43-Gln12	52.11	1.49
Asp15-Ile46	4.47	5.91	Asn61- Ser74	14.39	30.65	Gln12-His13	9.51	23.01
Ile46-His13	37.53	38.33	Ser74-Ser72	63.61	60.12	His13-Thr44	21.74	42.73
His13-Thr44 (Leu115)	21.74	22.50	Ser72-Asn102	2.29	3.30	Thr44-Gln117	23.51	44.17
Thr44 (Leu115)-Gln117	25.31	14.37	Asn102-Arg101	28.60	55.38	Gln117-Asp116	15.57	23.48
Gln117-Asp116	15.57	31.42	Arg101-Phe76	52.73	64.72	Asp116-His114	2.72	3.36
Asp116-His114	2.72	3.11	Phe76-Val78	1.49	2.93	His114-Ala106	27.33	32.27
His114-Ala106	27.33	70.68	Val78-Thr79	2.96	1.85	Ala106-Val113	43.10	61.57
Ala106-Val113	43.10	64.14	Thr79-Phe45*	62.73	83.37			

Italic font amino acid residues in parenthesis: Mutated residues.

Normal font amino acid residues in parenthesis: Amino acids appearing in hydrogen bond interaction path of mutants.

*Continuation of hydrogen bond interaction path.

We also examined the total hydrogen bond occupancy for the shortest path. The sum of hydrogen bond occupancy of all the pairs was 280.39 for K17I compared to 187.99 for WT-ANG (Table 1). The highest sum for hydrogen bond in L35P was 232.57. These results show that the higher hydrogen bond occupancy for His114-Ala106 and Ala106-Val113 is instrumental in shifting the dihedral angle from −80° to −179°. The hydrogen bond interaction networks for S28N and K60E mutants were also created. It was observed that S28N possesses a shortest path mediated through Leu115, while for K60E, it is mediated through Thr44 (Figures 7, 9; Figure S5).

Structural study reveals that Leu115 in ANG plays a similar functional role as Phe120 does in RNase A. The presence of Leu115 in ANG removes potential interactions with the pyrimidine ring and contributes to weaker ribonucleolytic activity [22]. Our results show that hydrogen bond interaction paths mediated through Leu115 are associated with conformational switching of His114 in mutants exhibiting loss of ribonucleolytic activity. It was also observed that the change in the dihedral angle of His114 was preceded by a realignment of Gln117. This realignment of Gln117 stabilizes the altered dihedral position of His114. The new orientation of Gln117 in the proximity of the catalytic triad obstructs the pyrimidine-binding site. The hydrogen bond between Thr44 and Thr80 has also been implicated in the loss of ribonucleolytic activity [22]. Crystal structure of ANG shows the existence of a hydrogen bond between Thr44 and Thr80, and Asp116 and Ser118 [22]. The weak ribonucleolytic activity compared to RNase A has been attributed to the presence of these hydrogen bonds. Since these hydrogen bonds may be altered in the mutants, we determined the percentage hydrogen bonding occupancy between Thr44 and Thr80 as well as Asp116 and Ser118 from MD trajectories. Percentage hydrogen bonding occupancy of WT-ANG and mutants has been presented (Table 2). We observed that the hydrogen bonding occupancy in case of K17I, S28N and L35P mutants were about 30% higher compared to WT-ANG, P112L, K60E and V113I mutants for Thr44-Thr80. For the Asp116-Ser118 hydrogen bond, the occupancy was about 20% higher for the K17I, S28N variants and L35P mutant.

**Table 2:** Percentage hydrogen bonding occupancy between Thr44-Thr80 and Asp116-Ser118.

ANG	H-bonding occupancy (%)	
	Thr44 — Thr80	Asp116 — Ser118
WT-ANG	51.38	62.18
K17I	96.87	95.31
S28N	82.51	74.27
P112L	53.66	69.24
L35P	84.59	89.36
K60E	49.92	61.76
V113I	50.36	63.15

#### Docking Analyses

**Figure 13:**
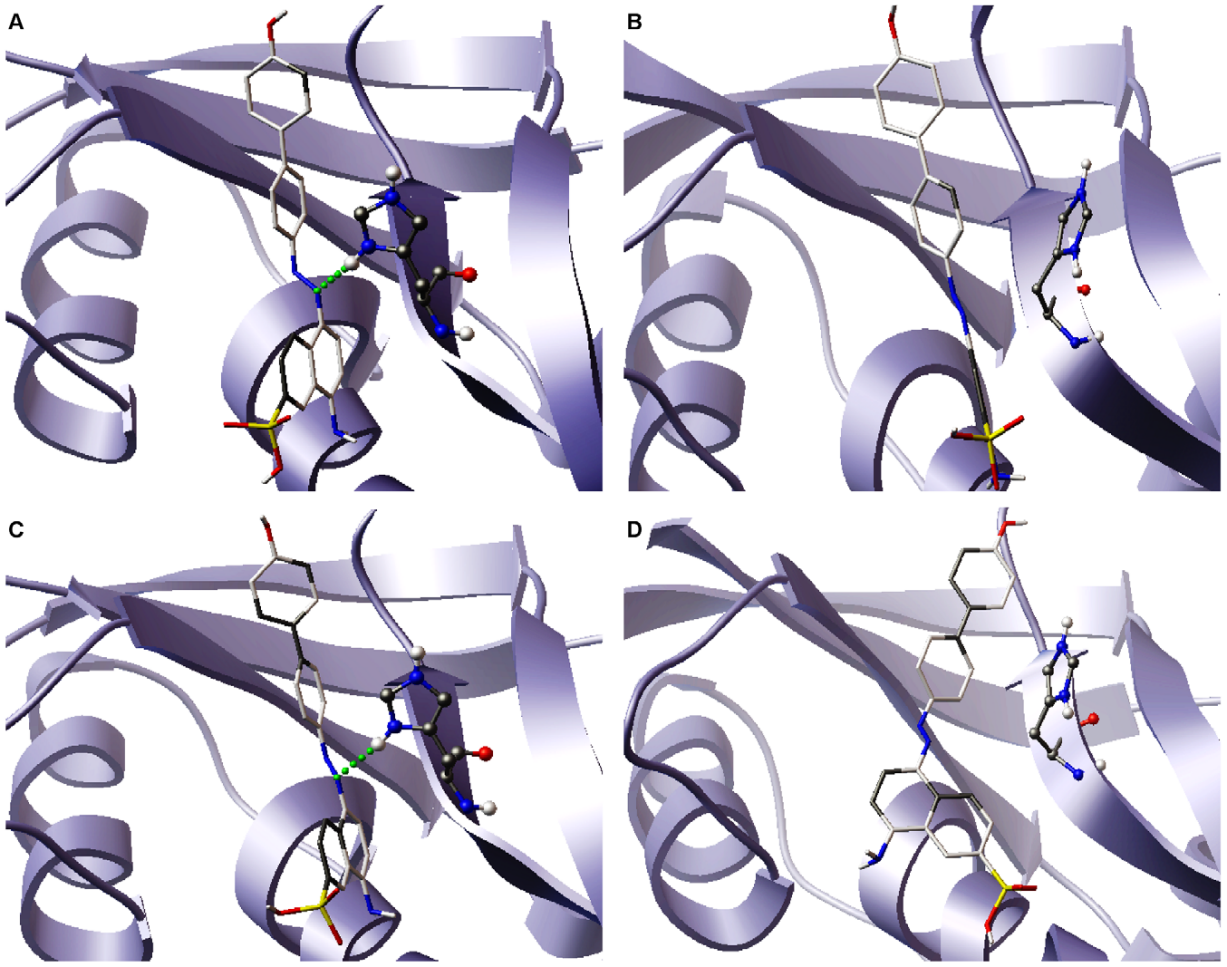
Lowest-energy AutoDock poses of NCI-65828 with His114 in K17I and L35P mutants. Stereo views of lowest-energy AutoDock poses of K17I and L35P mutants using NCI-65828. The backbone trace of ANG is shown along with the His114 residue as stick model depicting the hydrogen bond between the azo-group of the inhibitor and His114 (in green dashed line). In K17I, (A) presence hydrogen bond in native conformation with His114 (B) and its absence in the altered conformation of His114 were visualized. Similarly, in L35P, (C) presence hydrogen bond in native conformation with His114 (D) and its absence in the altered conformation of His114 are shown. Also shown is how the conformational switching of His114 affects substrate binding at the catalytic site.

As revealed from simulation, the dihedral angle of His114 in case of K17I variant and L35P mutant changes significantly and may result in the loss of ribonucleolytic activity. To confirm this, we carried out molecular docking simulations for WT-ANG and K17I, S28N, P112L, L35P, K60E and V113I mutants with an inhibitor of ribonucleolytic activity of angiogenin, NCI-65828. Snapshots of the native and altered His114 conformation were extracted from MD trajectories over 50 ns. These structures were used to carry out docking simulations and explain the loss of ribonucleolytic activity [38]. A strong interaction with WT-ANG and the inhibitor was observed as expected in the native conformation possessing ribonucleolytic activity (see Figure S6). Similarly, docking between the inhibitor and K17I, S28N, P112L, V113I variants and L35P, K60E mutants were performed using AutoDock [29], [30] and ParDOCK [32]–[34] and the binding energies for each case were determined and presented (Table 3). It was observed that the binding energy in those cases where the dihedral angle of the His114 changes significantly was lower compared to the WT-ANG conformation. In addition to this, the hydrogen-bond interactions for each docked conformation were examined. It was observed that the hydrogen bond between the azo-group of NCI-65828 and His114 HD1 exists only for WT-ANG catalytic triad conformation. In the case of K17I and L35P mutants, where there is a conformational switching of the His114, the hydrogen bond ceases to exist when the dihedral angle of His114 shifts by 99° (Figure 13). Binding orientation of NCI-65828 with K17I and L35P in the native and altered conformations is shown as surface model (see Figure S7).

**Table 3:** Comparison of AutoDock and ParDOCK binding energy scores for NCI-65828 complexes with WT-ANG and ANG mutants.

	AutoDock binding energy scores		ParDOCK binding energy scores	
ANG	Native conformation	Altered conformation	Native conformation	Altered conformation
WT-ANG*	−8.74 kcal/mol		−4.83 kcal/mol	
K17I	−7.72 kcal/mol	−4.84 kcal/mol	−3.33 kcal/mol	−2.44 kcal/mol
S28N	−6.28 kcal/mol	−4.46 kcal/mol	−3.87 kcal/mol	−3.00 kcal/mol
P112L	−7.66 kcal/mol	−4.38 kcal/mol	−3.49 kcal/mol	−2.99 kcal/mol
L35P	−7.04 kcal/mol	−4.63 kcal/mol	−3.57 kcal/mol	−1.97 kcal/mol
K60E*	−7.18 kcal/mol		−4.41 kcal/mol	
V113I*	−7.44 kcal/mol		−3.80 kcal/mol	

*(ANG in which there is no conformational change of the catalytic residue His114).

#### Loss of Nuclear Translocation Activity

**Figure 14:**
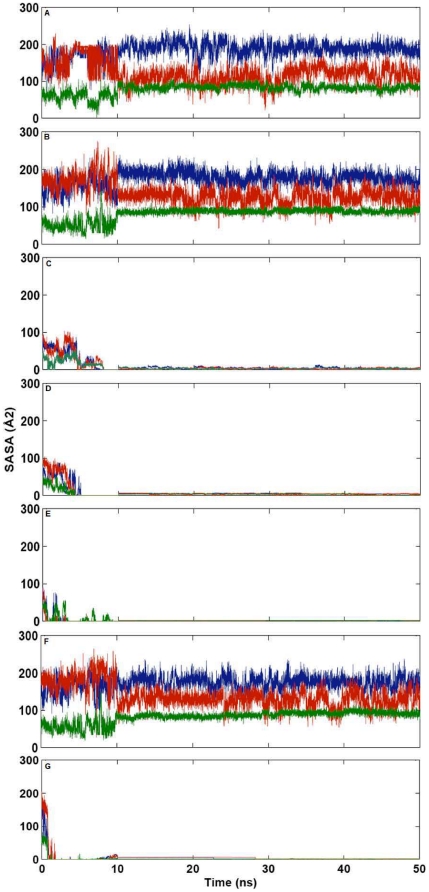
Computed change in solvent accessible surface area for WT-ANG and mutants. Variation of solvent accessible surface area (SASA) of nuclear localization signal residues R31, R32 and R33 over the period of simulation (A) WT-ANG (B) K17I (C) S28N (D) P112L (E) L35P (F) K60E and (G) V113I. (R31: blue, R32: red and R33: green).

**Figure 15:**
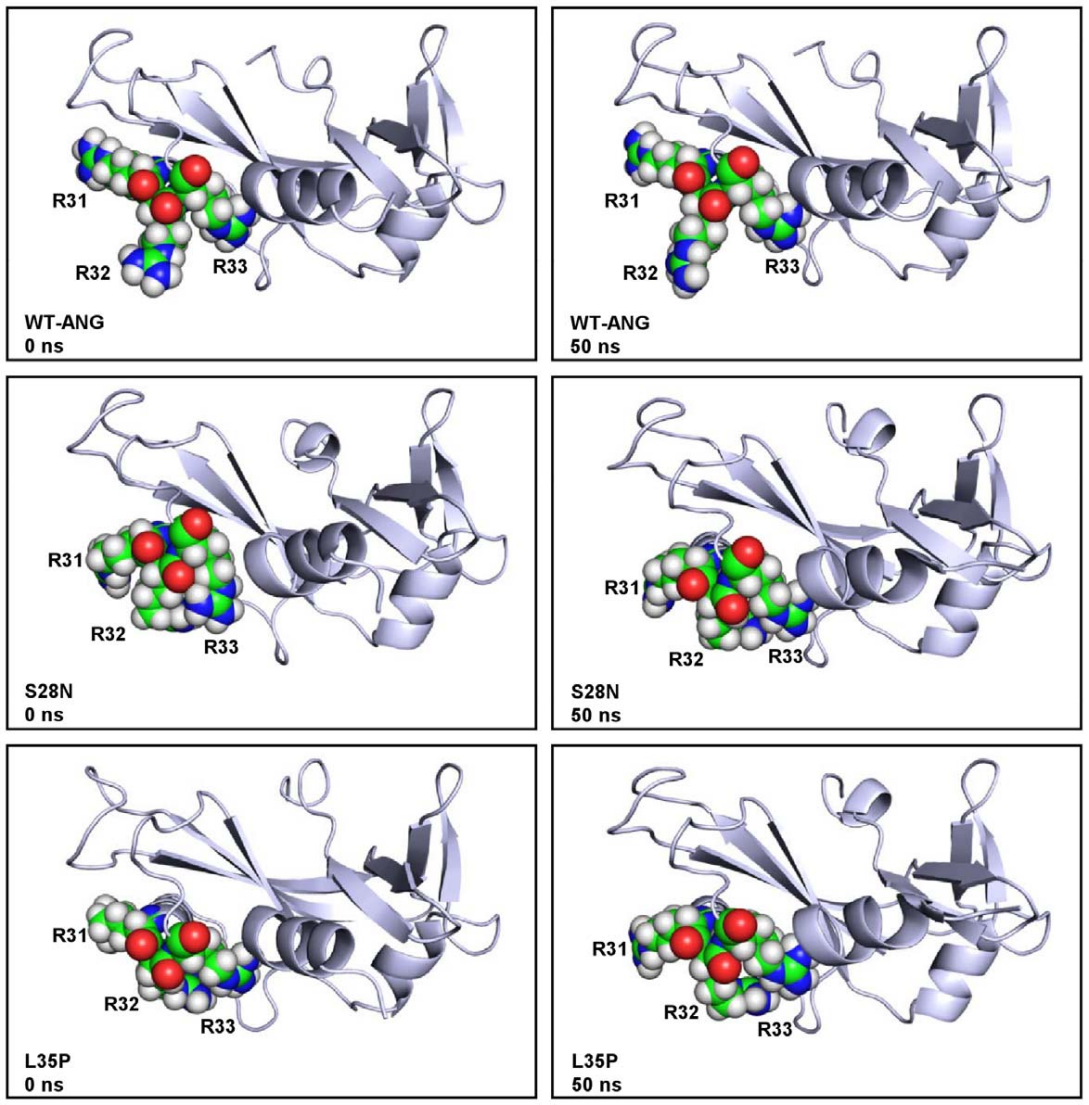
Changes in folding of residues ^31^RRR^33^ for WT-ANG, S28N and L35P mutants. Comparison of the changes in the folding of the residues R31, R32 and R33 at initial condition and at 50 ns of simulation. The residues are in open conformation for WT-ANG throughout the duration of simulation. For S28N and L35P mutants, a closed conformation is dominant. Figure produced using PyMOL [53].

Wu et al. [8] reported S28N and P112L mutations in ANG obtained from North American ALS cohort. Through their functional assay experiments, they established that these mutations in ANG resulted in loss of nuclear translocation activity. Since nuclear localization signal of ANG is exposed on the protein surface and largely accessible to solvent, the effect of change in the solvent accessible surface area (SASA) on nuclear translocation activity was investigated. SASA of the nuclear localization signal residues (^31^RRR^33^) was calculated for each mutant and compared with WT-ANG. SASA was determined for 50 ns for Arg31, Arg32 and Arg33, the three successive Arginine residues known to play a pivotal role in nuclear translocation activity (Figure 14). The average SASA over successive 10 ns time intervals was calculated for each mutant and WT-ANG. It was observed that SASA values for S28N, P112L and L35P mutants stabilized after 10 ns, while the others stabilized after 30 ns (see Figure S8). Considering the last 10 ns duration, we noticed that the variants S28N and P112L have SASA below 15 Å^2^ compared to WT-ANG and K17I, which possessed SASA above 385 Å^2^ (Figure 14). This difference of more than 350 Å^2^ correlated with experimentally determined loss of nuclear translocation activity [8]. We also observed that the SASA for L35P and V113I mutants were even lower. However, K60E had a SASA of 388 Å^2^. In addition, the snapshots of nuclear localization signal residues obtained at 50 ns revealed that ^31^RRR^33^ was loosely packed for WT-ANG and mutants exhibiting no loss of nuclear translocation activity, while it was tightly packed for mutants exhibiting loss of nuclear translocation activity (Figure 15). The volume of the smallest sphere in which the residues ^31^RRR^33^ can fit was computed for the entire period of simulation and is shown (see Figure S9). It was observed that average sphere volume for WT-ANG, K17I and K60E, which exhibited no loss of nuclear translocation activity, was greater than 2502 Å^3^. The mutations L35P, S28N, P112L and V113I, which exhibited loss of nuclear translocation activity had average sphere volumes less than 804 Å^3^ (Table 4).

**Table 4:** SASA values of R31, R32 and R33 over last 10 ns and calculated smallest equivalent sphere volume for WT-ANG and ANG mutants.

	SASA (Å^2^)				Volume (Å^3^)
ANG	R31	R32	R33	Total	
WT-ANG	187.72	122.09	81.35	391.16	2628.83
K17I	175.36	126.76	87.48	389.61	2502.31
S28N	3.19	2.65	2.42	8.26	783.04
P112L	4.79	3.93	2.74	11.46	717.44
L35P	1.13	1.27	1.24	3.66	784.39
K60E	175.69	128.06	84.50	388.25	2753.69
V113I	1.58	1.21	1.06	3.86	804.55

#### F-SNP database prediction for L35P and K60E mutants

We have verified using F-SNP database [39] (http://compbio.cs.queensu.ca/F-SNP/) that T195C SNP encoding L35P mutant would have a detrimental effect on ANG and is likely to cause ALS. F-SNP computes a functional significance (FS) score [40], using a tool reliability (TR) score based on Bayes' rule and information obtained from 16 bioinformatics tools and databases. For L35P mutant, the FS score obtained was 0.764 and classified as deleterious. Similarly, the FS score obtained for A238G SNP encoding K60E mutant was 0.533 and classified as benign. This further supports our result that T195C SNP encoding L35P mutant would be correlated with ALS.

## Discussion

Through the results of this investigation, we have established that the conformational switching of His114 of the catalytic triad of ANG is responsible for the loss of ribonucleolytic activity. We calculated the changes in the dihedral angle, performed hydrogen bond interaction analyses and carried out docking simulations with an inhibitor to ascertain this. We first established that our observations were in line with the clinical and experimental findings in the literature. We built our discussion around K17I variant and L35P mutant. Using the results of K17I as a basis, we predict that L35P may also exhibit loss of angiogenic activity and its association with ALS.

### Loss of Ribonucleolytic Activity

The loss of ribonucleolytic activity of ANG mutants was examined by scanning through the snapshots of the catalytic triad at various time instances during VMD visualization. We observed that among the residues His13, Lys40 and His114, only His114 exhibited dramatic changes in conformation relative to the positions of the other residues. Therefore, the HA-CA-CB-CG dihedral angle of His114 was measured for the whole duration of simulation for WT-ANG and all the mutants. Snapshots were taken at 10 ns intervals and correlated with the change in the dihedral angles compared to the native position in WT-ANG (Figure 4, Figures S3 and S4). The observed loss in ribonucleolytic activity varied from severe in K17I, S28N and L35P to mild in P112L and insignificant in K60E and V113I, and this correlated positively with the frequency with which the dihedral angle changed from −80° to −179° (Figures 4, 5; Figures S3 and S4).

Our observations find support in the work of Wu et al. [8], who identified four mutations in the coding region of *ANG* from 298 ALS patients. They showed using functional assays that these mutants exhibited a complete loss of angiogenic activity due to either loss of ribonucleolytic activity, loss of nuclear translocation activity or both. They reported that K17I variant had lost more than 95% ribonucleolytic activity, whereas, S28N and P112L had lost nuclear translocation activity, although, S28N had 9% and P112L had partial ribonucleolytic activity. The importance of each of the residues of the catalytic triad, His13, Lys40 and His114, had been established by a series of site directed mutagenesis work carried out by Shapiro's group. They observed a significant decrease in ribonucleolytic activity by replacing His13 or His114 by Ala [41] or Lys40 by Gln [42]. Since His114 is the more important residue of the catalytic triad, the H114N mutant exhibited a 3300 fold reduction in activity [41]. Our simulation results also show that it is only the His114, which exhibits conformational switching in mutants exhibiting loss of ribonucleolytic activity. Subsequent crystal structure studies (2.0 Å) of two of these active sites of the catalytically inactive variants K40Q and H13A, showed no significant changes outside the catalytic site. Loss of angiogenicity was attributed to the disruption of the catalytic triad [22]. Our RMSD and RMSF results also did not show any major difference between the mutants and WT-ANG (Figure 3). Consequently, the question arises as to how does a mutation distal from the active site affects ribonucleolytic activity because the resulting loss in ribonucleolytic activity is not intuitive from the crystal structure.

To understand how this may happen, we first examined the hydrogen bond interaction networks for K17I, S28N, L35P and K60E mutants. We identified based on a 3.2 Å cut-off, a shortest path by which contiguous hydrogen bonds may exercise influence from the site of mutation to His114 (Figures 6, 7, 8, 9). The hydrogen bond occupancy of each adjacent amino acid pair appearing in this path was computed. For all cases where conformational switching of His114 was observed in the simulations, there was a significant increase in hydrogen bond occupancies between His114-Ala106 and Ala106-Val113. Further, it was observed that the shortest path was conserved for mutants exhibiting loss of ribonucleolytic activity during conformational switching of His114; while other paths leading from the site of mutation to His114 lost continuity during reorientation of His114. del Sol et al. [43] have also identified conserved residues that occur in shortest paths and play a vital role in long range interactions and allosteric communication in seven protein families. Therefore, the path traced by tertiary hydrogen bonds is an important characteristic that may be used to infer how a perturbation caused by a mutation exerts influence on a distant residue [44]. Similar graph-theoretic studies have been carried out to understand structure-function relationships of proteins and how amino acid residues act as nucleation centers for protein folding [43], [45]. We have presented the hydrogen bond occupancies of amino acid pairs occurring in the shortest paths for K17I, S28N, L35P and K60E mutants in the order of increasing ribonucleolytic activity in Table 1. Although, loss of ribonucleolytic activity has been demonstrated in K17I [8], this is not the case for L35P. In a SNP genotype distribution study carried out across six populations: Japanese (n = 167), Korean (n = 90), Mongolian (n = 92), Ovambos (n = 86), Turkish (n = 87), and German (n = 70), correlation between clinical and genetic data for L35P, K60E and H76Q could not be established because of limited allele frequency data [21]. We compared the results obtained for K17I and L35P. Considering the RMSD, RMSF, dihedral angle change and hydrogen bond occupancies, we predict that L35P will exhibit loss of angiogenic activity by the loss of ribonucleolytic activity.

The crystal structure study by Leonidas et al. [22] also established that the obstruction of the pyrimidine-binding site by Gln117, the existence of a hydrogen bond between Thr44-Thr80 and Asp116-Ser118, were structural weak points of ANG but not found in other RNase A counterparts. We determined the hydrogen bond occupancies for Thr44-Thr80 and Asp116-Ser118 for all mutants (Table 2). The hydrogen bond occupancy is highest for K17I variant (96.87% and 95.31%, respectively) followed by L35P mutant (84.59% and 89.36%, respectively). The occupancy values were lower in mutants retaining ribonucleolytic activity. Site directed mutagenesis experiments carried out by Harper and Vallee [46] showed that D116N, D116A and D116H mutants had higher ribonucleolytic activity. The mutation probably reorients the water structure associated with the side chain of His114, creating a more favorable conformation for catalysis. Therefore, the higher H-bond occupancies of K17I and L35P, confirms that the presence of H-bond through Asp116, plays an important role in the loss of ribonucleolytic activity [46]. In addition, a study of the mutants of Thr44, reported in the literature, T44A, T44H and T44D, had shown conclusively the importance of Thr44 for angiogenic activity [47]. T44H, which is 2–4% active in cleaving polynucleotides, has at least 100 times less angiogenic activity. In a subsequent study, it was established that the Thr44-Thr80 hydrogen bond attenuates ribonucleolytic activity of ANG [48]. Through their study of the T44A/T80A and Q117A/T80A double mutants, they demonstrated that the changes in potency and specificity were mediated by Thr44.

In addition to this, the hydrogen-bond interactions for each docked conformation were examined. It was observed that the hydrogen bond between the azo-group of NCI-65828 and His114 HD1 exists only for WT-ANG catalytic triad conformation. For K17I and L35P, where there is a conformational switching of the His114, the hydrogen bond ceases to exist when the dihedral angle of His114 shifts by 99° (Figure 13). The binding energies computed using AutoDock and ParDOCK both show consistent reduction commensurate with the reorientation of His114 by 99° (Table 3).

### Loss of Nuclear Translocation Activity

Angiogenesis involves activation of endothelial cells and basement membrane degradation, followed by translocation, proliferation, and differentiation of the endothelial cells into capillary structures. In one of the early investigations, it was demonstrated that nuclear translocation of ANG was critical for angiogenesis. Moroianu and Riordan [49], [50] showed using immunoflorescence microscopy that the R33A mutant was not internalized in proliferating calf pulmonary artery endothelial (CPAE) cells. This established that the stretch of three arginines, ^31^RRR^33^, was part of the nuclear localization signal. At the same time, two enzymatically inactive mutants, K40Q and H13A, translocated to the nucleoli. Not all the mechanisms of actions of ANG are well understood [51], [52]. We have tried to understand why certain mutants lost their nuclear translocation activity by examining the changes in SASA. The SASAs for R31, R32 and R33, were computed over the course of simulation for each mutant. It was observed that S28N and P112L variants that had lost nuclear translocation activity, possessed distinctly lower SASA compared to K17I and K60E, which retained nuclear translocation activity (Figure 14). As the simulation progressed, the R31, R32 and R33 residues, folded in a manner that was inaccessible to the solvent (Figure 15). In V113I this happens within 2 ns of the simulation; for S28N, P112L and L35P, this happens within 10 ns. The average SASA over the last 10 ns for ^31^RRR^33^ was calculated for each mutant from their respective MD trajectories. These values were summed together and compared (Table 4). It was observed that for S28N and P112L, for which the loss of nuclear translocation activity has been confirmed experimentally [8], the total SASA was 8.26 Å^2^ and 11.46 Å^2^ respectively. For the V113I variant identified in Italian ALS patients [16], the total SASA was 3.86 Å^2^. Val113 is highly conserved during evolution but located within the hydrophobic core of ANG. Therefore, its role in angiogenesis is not clearly defined [16]. Our results indicate that V113I is implicated in ALS probably by loss of nuclear translocation activity. Since the lowest value of 3.66 Å^2^ was obtained for L35P, our model indicates that this mutant may be linked to ALS.

The other mutations K17I and K60E, which did not exhibit loss of nuclear translocation activity, had a total SASA of 389.61 Å^2^ and 388.25 Å^2^, respectively over the last 10 ns duration and compared well with the value of 391.16 Å^2^ for WT-ANG (see Figure S8). The average volume calculated over 50 ns duration of the smallest sphere that can enclose these three residues together is also given in Table 4. The diameter was taken as the maximum distance between the extremities of R31 and R33. The calculated volume is distinctly lower for mutations exhibiting a loss of nuclear translocation activity (see Figure S9).

The predictions of our model were also supported by the results obtained from F-SNP database [39], [40] (http://compbio.cs.queensu.ca/F-SNP/) which classified T195C SNP encoding L35P mutant as deleterious and A238G SNP encoding K60E as benign. Our model indicates that L35P may exhibit a loss of ribonucleolytic activity and nuclear translocation activity. However, in the absence of experimental studies, further genetic data will be required to support our prediction about the role of L35P mutant in ALS pathogenesis.

## Supporting Information

Figure S1 **Comparison of backbone RMSF values of crystal structure and simulated structure.** Comparison of RMSF values of the backbone atoms calculated from the crystallographic temperature factors (black line) and obtained from MD simulation (red line) at 300 K, as a function of residue number.
(TIF)

Figure S2 **Computed dihedral angle change of His114 of WT-ANG in its mutant conformation.** The HA-CA-CB-CG dihedral angle change of catalytic residue His114 computed as a function of time after rotating His114 about 99° similar to that of the mutant conformation. His114 acquires its native conformation within 1 ns time interval and stabilizes thereafter.
(TIF)

Figure S3 **Conformational switching of catalytic residue His114 in S28N and P112L variants.** Reorientation of the catalytic triad residue His114 at a regular interval of 10 ns over 50 ns time period during the MD simulation of S28N and P112L variants. In these figures, T = 0 ns is the time when the temperature of the system has been maintained at 300 K. Figure produced using PyMOL [53].
(TIF)

Figure S4 **Conformational switching of catalytic residue His114 in K60E and V113I mutants.** Reorientation of the catalytic triad residue His114 at a regular interval of 10 ns over 50 ns time period during the MD simulation of K60E and V113I mutants. In these figures, T = 0 ns is the time when the temperature of the system has been maintained at 300 K. Figure produced using PyMOL [53].
(TIF)

Figure S5 **Residues interacting through hydrogen bonds from the site of mutation to His114 in S28N variant.** Ribbon representation of S28N mutant angiogenin, residues involved in the hydrogen bonding connected from the site of mutation to catalytic residue His114 have been shown in stick model and represented as marine blue color. Catalytic triad residues have been shown as stick model and represented in green color. Hydrogen bonds between residues are shown in red dotted lines. Figure produced using PyMOL [53].
(TIF)

Figure S6 **Lowest-energy AutoDock poses of NCI-65828 with His114 in WT-ANG.** Stereoviews of lowest-energy AutoDock poses of WT-ANG. The backbone trace of ANG is shown along with the His114 residue as stick model. Predicted hydrogen bond before conformational switching of His114 is shown as dashed lines (green color).
(TIF)

Figure S7 **Surface view of Lowest-energy AutoDock poses of NCI-65828 with His114 in K17I and L35P.** Binding orientation of NCI-65828 represented as stick model predicted by the AutoDock Lamarckian Genetic Algorithm. ANG and His114 are shown as surface model. (A) Predicted hydrogen bond in native conformation of His114 is shown as dashed lines (green color) and sphere in K17I. (B) No hydrogen bond formed in altered conformation of His114 in K17I. (C) Predicted hydrogen bond in native conformation of His114 is shown as dashed lines (green color) and sphere in L35P mutant. (D) No hydrogen bond formed in altered conformation of His114 in L35P mutant.
(TIF)

Figure S8 **Average SASA values of nuclear localization signal residues ^31^RRR^33^ for WT-ANG and mutants.** The bar plot shows calculated average SASA of nuclear localization signal residues ^31^RRR^33^ over successive 10 ns time intervals for WT-ANG and mutants. SASA values of all the ANG forms were stable after 30 ns.
(TIF)

Figure S9 **Computed volume of nuclear localization signal residues ^31^RRR^33^ for WT-ANG and mutants.** Calculated volume of the nuclear localization signal residues ^31^RRR^33^ between WT-ANG and the mutants throughout the course of simulations at 300 K. WT-ANG, K17I, S28N, P112L, L35P, K60E, V113I are represented in black, red, dark green, blue, orange, pink, and light green, respectively.
(TIF)
