# Supplementary figures and images for: Mechanisms of Loss of Functions of Human Angiogenin Variants Implicated in Amyotrophic Lateral Sclerosis
Source: PLoS One. 2012 Feb 27;7(2):e32479. doi: 10.1371/journal.pone.0032479 (PMC3288110; doi:10.1371/journal.pone.0032479)

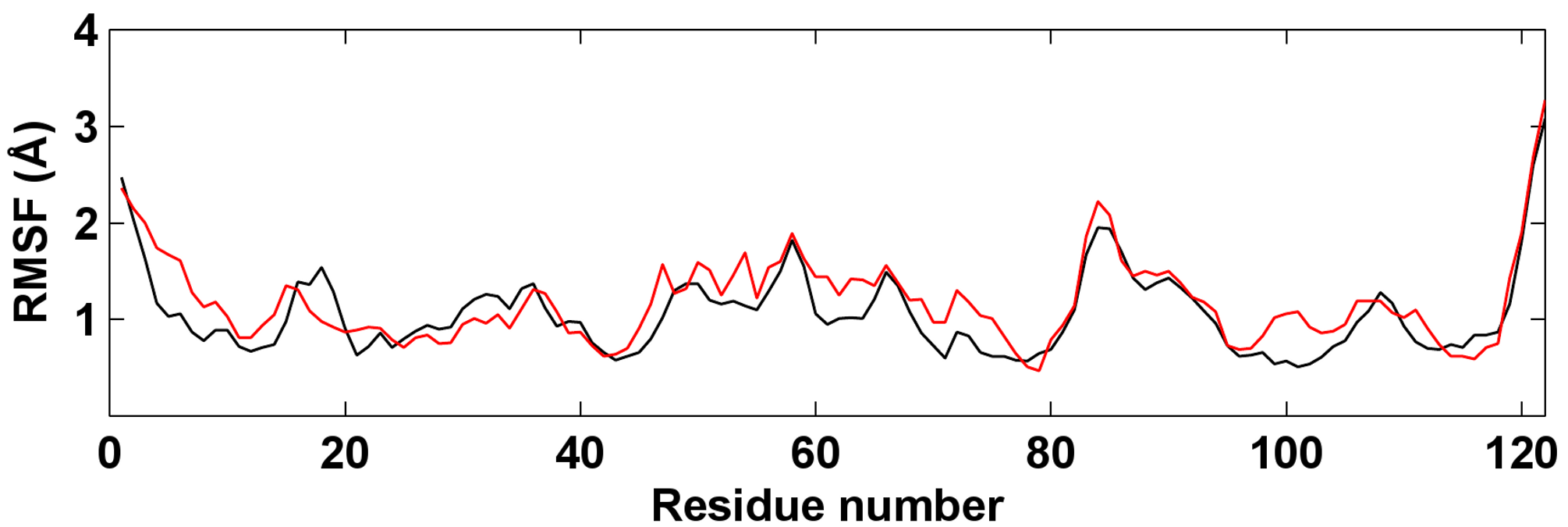

Supplement: Figure S1 — Comparison of backbone RMSF values of crystal structure and simulated structure. Comparison of RMSF values of the backbone atoms calculated from the crystallographic temperature factors (black line) and obtained from MD simulation (red line) at 300 K, as a function of residue number. (TIF) [file pone.0032479.s001.tif]

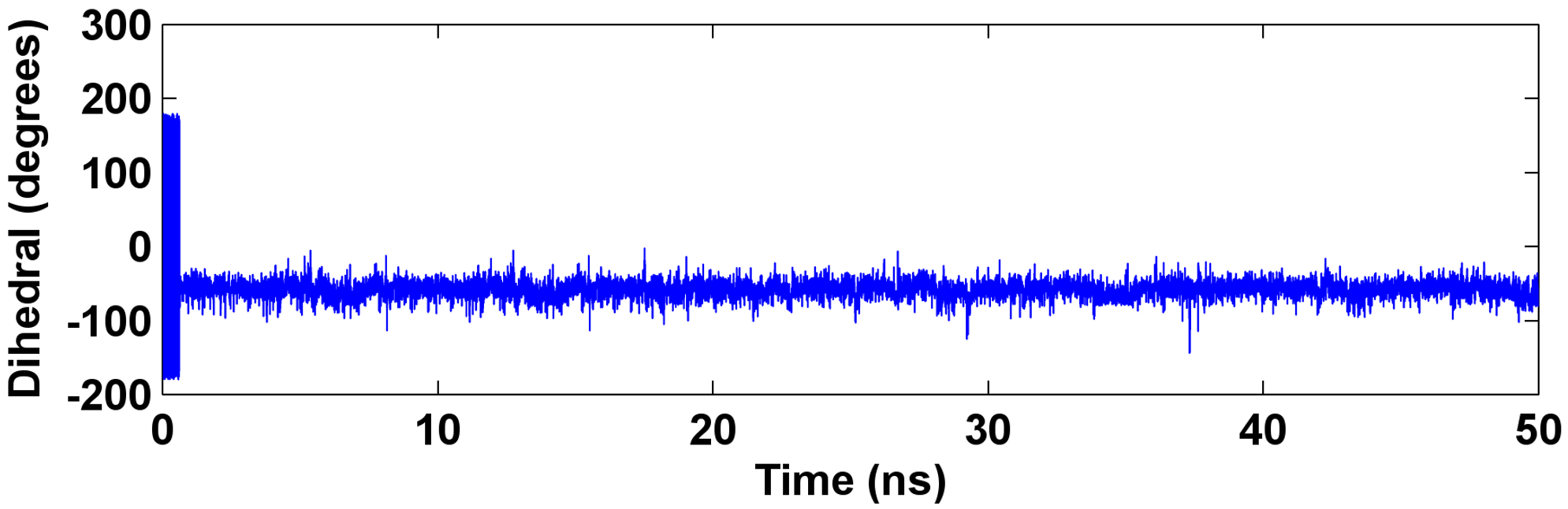

Supplement: Figure S2 — Computed dihedral angle change of His114 of WT-ANG in its mutant conformation. The HA-CA-CB-CG dihedral angle change of catalytic residue His114 computed as a function of time after rotating His114 about 99° similar to that of the mutant conformation. His114 acquires its native conformation within 1 ns time interval and stabilizes thereafter. (TIF) [file pone.0032479.s002.tif]

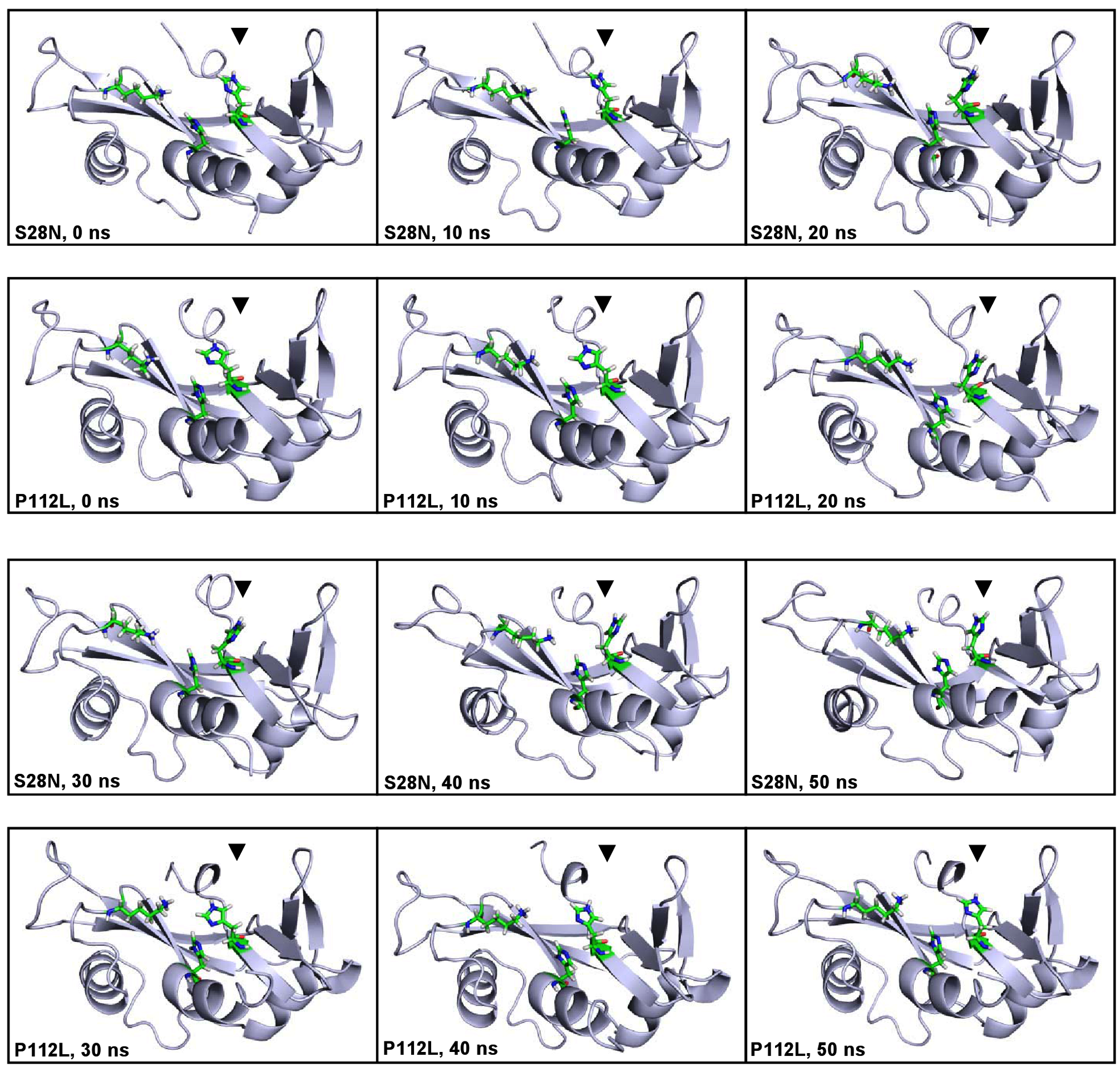

Supplement: Figure S3 — Conformational switching of catalytic residue His114 in S28N and P112L variants. Reorientation of the catalytic triad residue His114 at a regular interval of 10 ns over 50 ns time period during the MD simulation of S28N and P112L variants. In these figures, T = 0 ns is the time when the temperature of the system has been maintained at 300 K. Figure produced using PyMOL [53]. (TIF) [file pone.0032479.s003.tif]

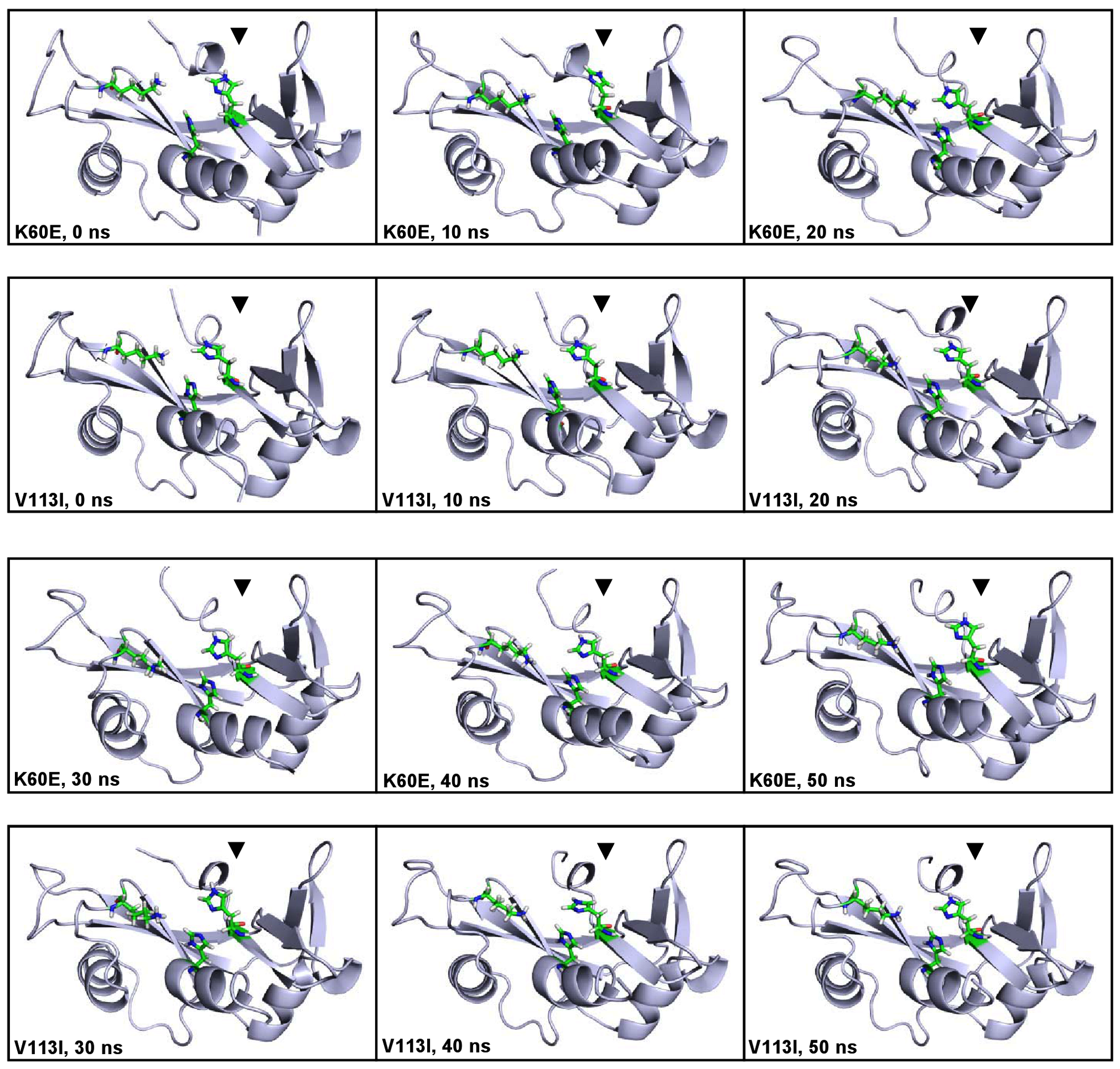

Supplement: Figure S4 — Conformational switching of catalytic residue His114 in K60E and V113I mutants. Reorientation of the catalytic triad residue His114 at a regular interval of 10 ns over 50 ns time period during the MD simulation of K60E and V113I mutants. In these figures, T = 0 ns is the time when the temperature of the system has been maintained at 300 K. Figure produced using PyMOL [53]. (TIF) [file pone.0032479.s004.tif]

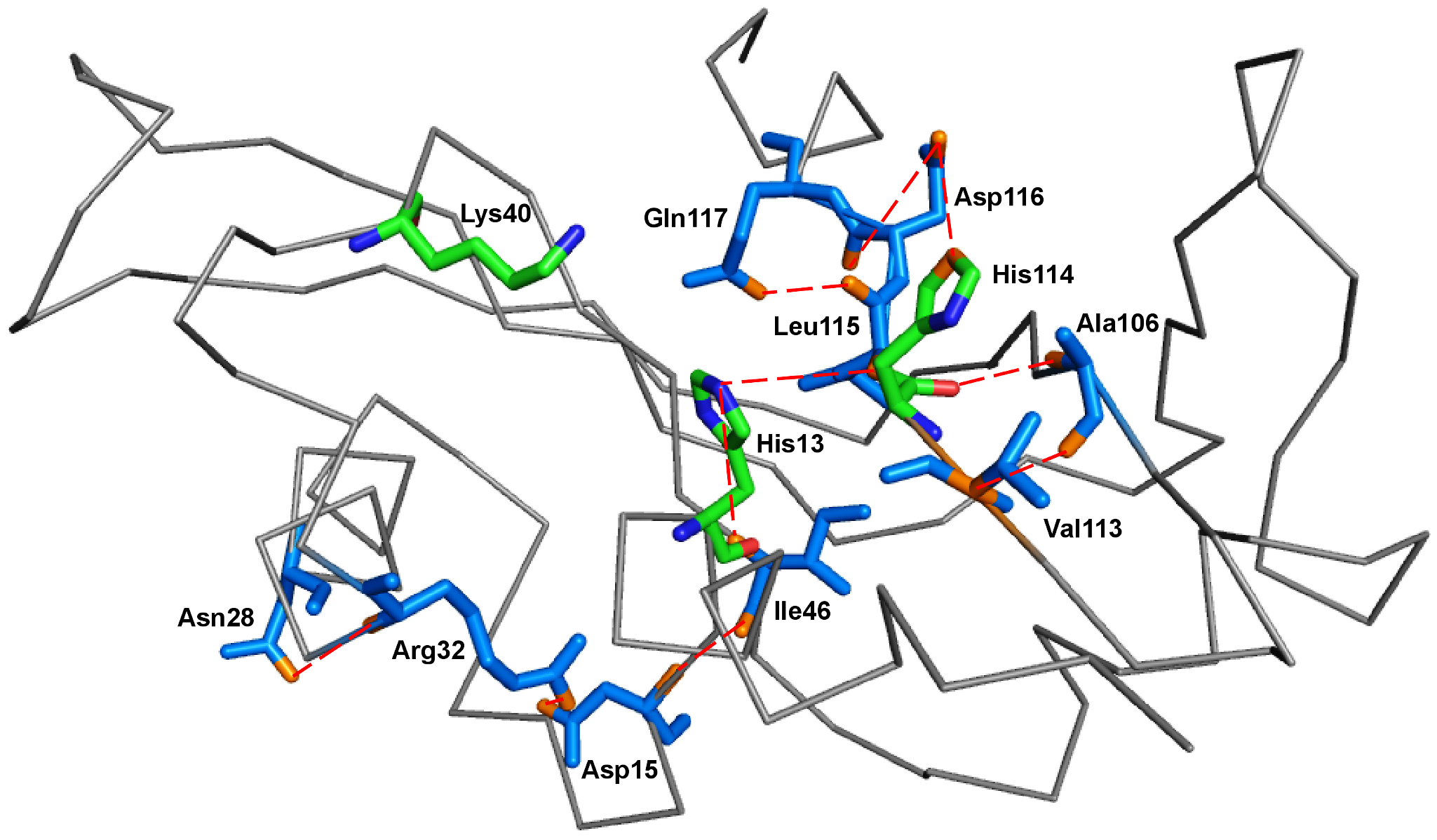

Supplement: Figure S5 — Residues interacting through hydrogen bonds from the site of mutation to His114 in S28N variant. Ribbon representation of S28N mutant angiogenin, residues involved in the hydrogen bonding connected from the site of mutation to catalytic residue His114 have been shown in stick model and represented as marine blue color. Catalytic triad residues have been shown as stick model and represented in green color. Hydrogen bonds between residues are shown in red dotted lines. Figure produced using PyMOL [53]. (TIF) [file pone.0032479.s005.tif]

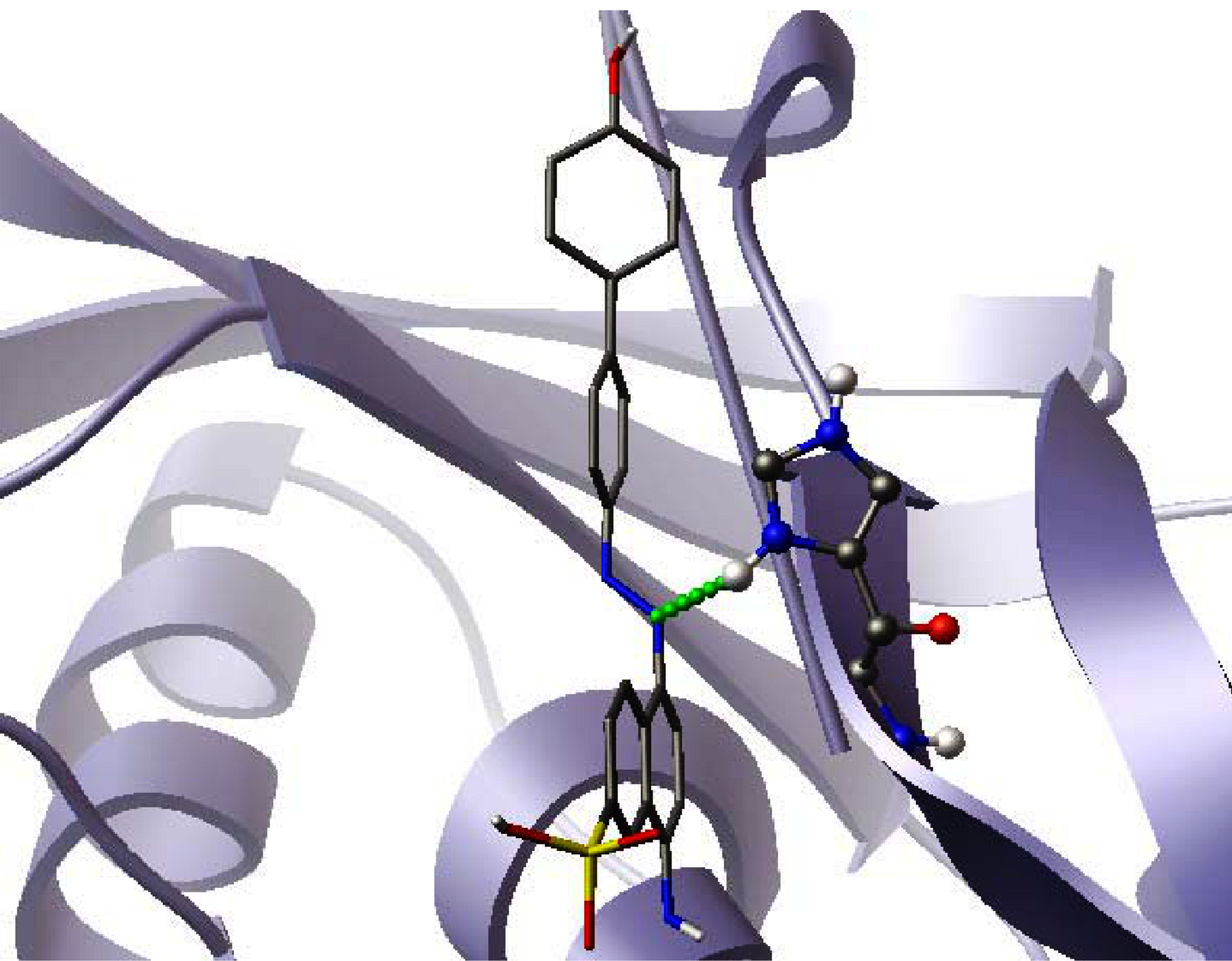

Supplement: Figure S6 — Lowest-energy AutoDock poses of NCI-65828 with His114 in WT-ANG. Stereoviews of lowest-energy AutoDock poses of WT-ANG. The backbone trace of ANG is shown along with the His114 residue as stick model. Predicted hydrogen bond before conformational switching of His114 is shown as dashed lines (green color). (TIF) [file pone.0032479.s006.tif]

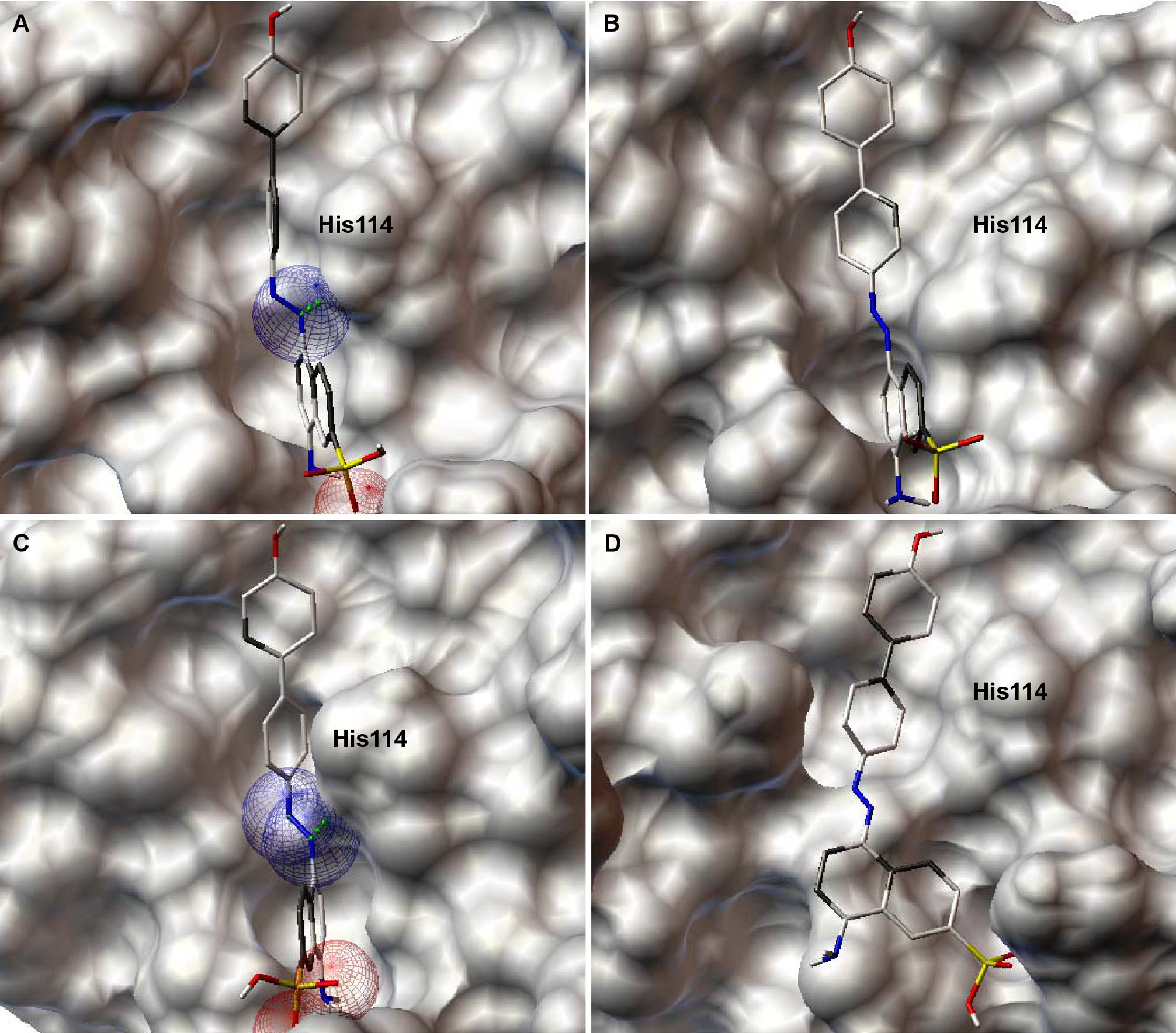

Supplement: Figure S7 — Surface view of Lowest-energy AutoDock poses of NCI-65828 with His114 in K17I and L35P. Binding orientation of NCI-65828 represented as stick model predicted by the AutoDock Lamarckian Genetic Algorithm. ANG and His114 are shown as surface model. (A) Predicted hydrogen bond in native conformation of His114 is shown as dashed lines (green color) and sphere in K17I. (B) No hydrogen bond formed in altered conformation of His114 in K17I. (C) Predicted hydrogen bond in native conformation of His114 is shown as dashed lines (green color) and sphere in L35P mutant. (D) No hydrogen bond formed in altered conformation of His114 in L35P mutant. (TIF) [file pone.0032479.s007.tif]

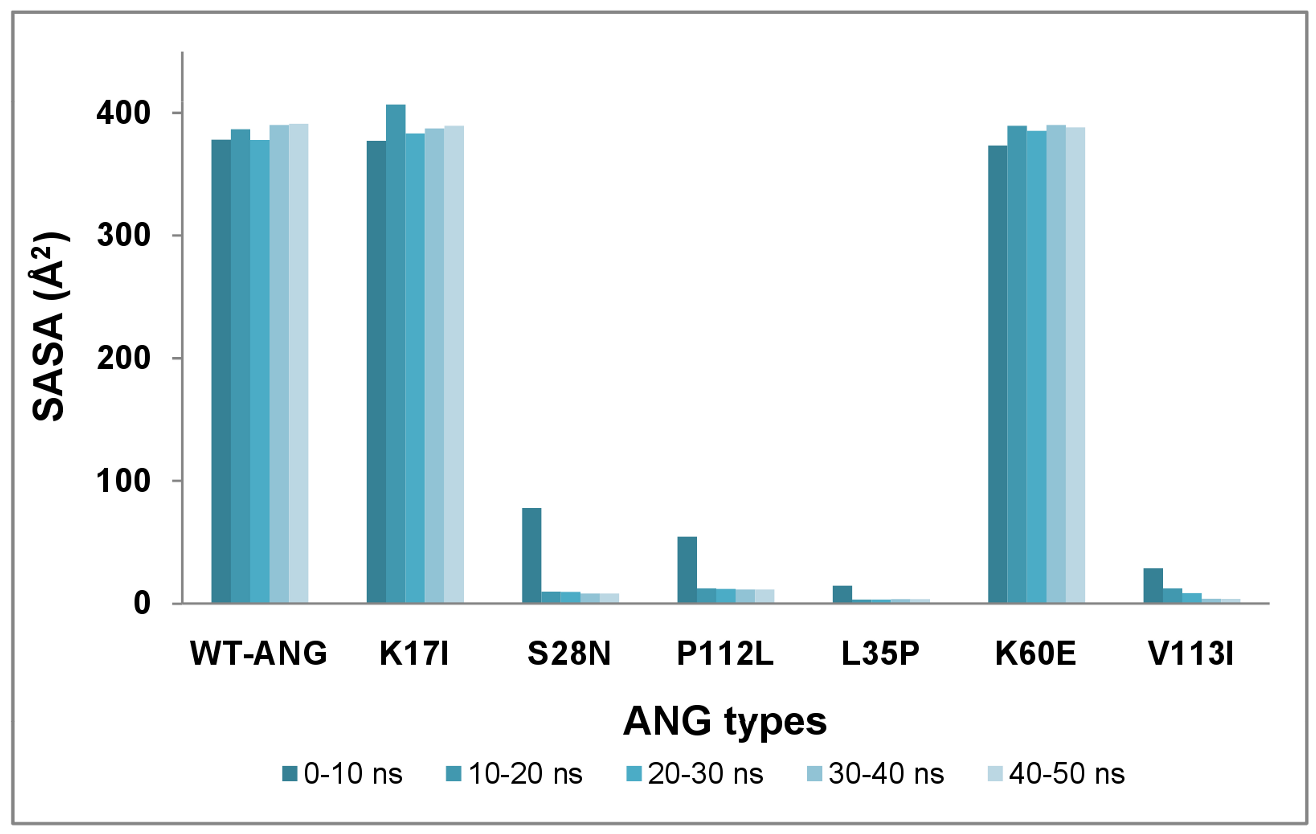

Supplement: Figure S8 — Average SASA values of nuclear localization signal residues 31RRR33 for WT-ANG and mutants. The bar plot shows calculated average SASA of nuclear localization signal residues 31RRR33 over successive 10 ns time intervals for WT-ANG and mutants. SASA values of all the ANG forms were stable after 30 ns. (TIF) [file pone.0032479.s008.tif]

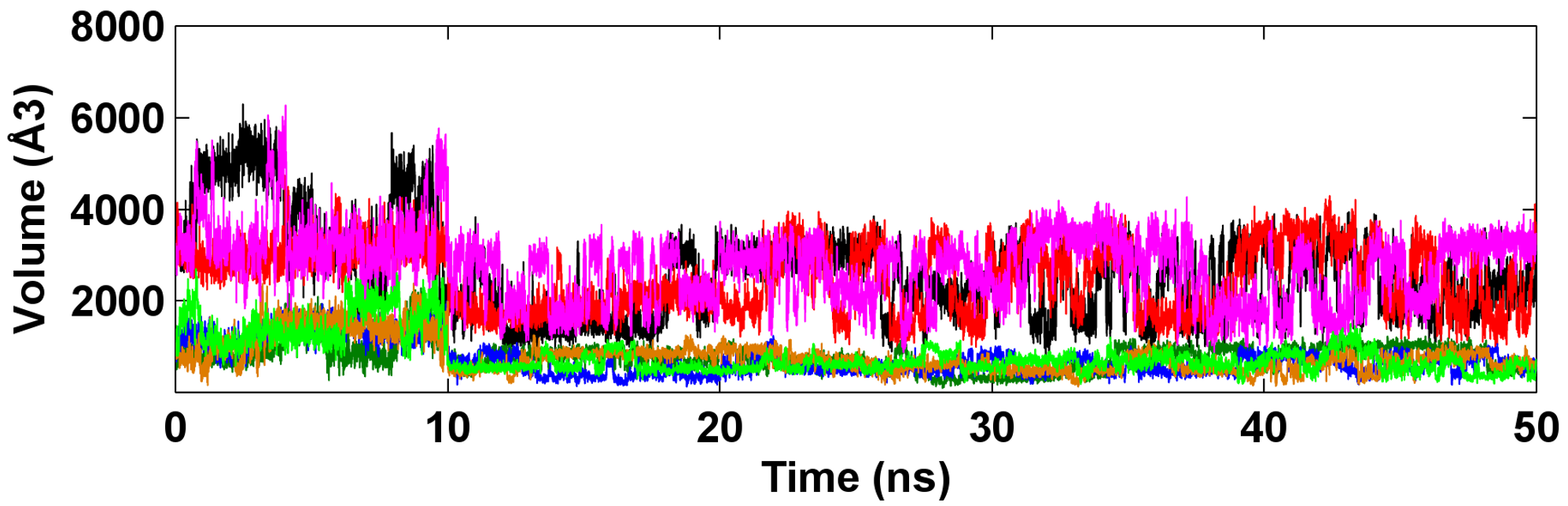

Supplement: Figure S9 — Computed volume of nuclear localization signal residues 31RRR33 for WT-ANG and mutants. Calculated volume of the nuclear localization signal residues 31RRR33 between WT-ANG and the mutants throughout the course of simulations at 300 K. WT-ANG, K17I, S28N, P112L, L35P, K60E, V113I are represented in black, red, dark green, blue, orange, pink, and light green, respectively. (TIF) [file pone.0032479.s009.tif]
